# Fed- and Fasted-State Performance of Pretomanid Amorphous Solid Dispersions Formulated with an Enteric Polymer

**DOI:** 10.1021/acs.molpharmaceut.3c00174

**Published:** 2023-05-23

**Authors:** Hanh Thuy Nguyen, Tu Van Duong, Sarah Jaw-Tsai, Rebecca Bruning-Barry, Poonam Pande, Rajneesh Taneja, Lynne S. Taylor

**Affiliations:** † Department of Industrial and Physical Pharmacy, College of Pharmacy, Purdue University, West Lafayette, Indiana 47907, United States; ‡ Sarah Jaw-Tsai Consulting Services, 12279 Skyracer Drive, Las Vegas, Nevada 89138, United States; § Global Health Technologies Program, RTI International, Research Triangle Park, North Carolina 27704, United States; ∥ Global Alliance for TB Drug Development (TB Alliance), 80 Pine Street, 20th Floor, New York, New York 10005, United States

**Keywords:** pretomanid, HPMCAS, enteric polymer, amorphous solid dispersion, crystallization, fasted state, fed state, food impact

## Abstract

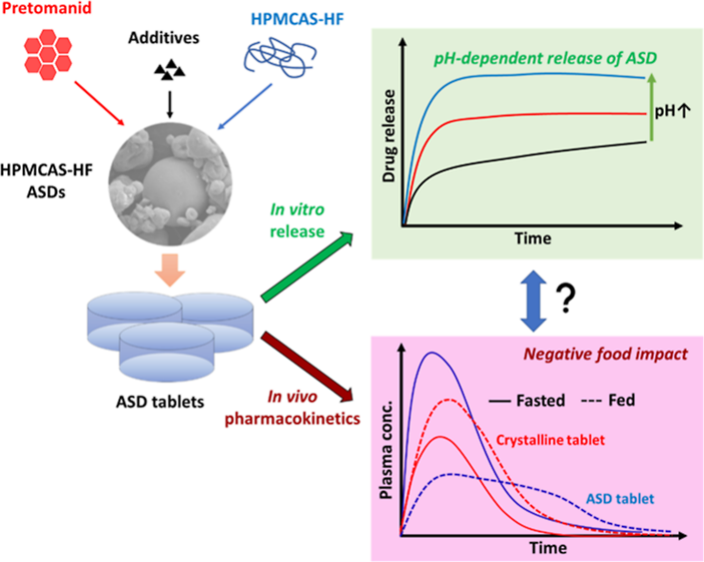

Weakly acid polymers with pH-responsive solubility are being used with increasing frequency in amorphous solid dispersion (ASD) formulations of drugs with low aqueous solubility. However, drug release and crystallization in a pH environment where the polymer is insoluble are not well understood. The aim of the current study was to develop ASD formulations optimized for release and supersaturation longevity of a rapidly crystallizing drug, pretomanid (PTM), and to evaluate a subset of these formulations in vivo. Following screening of several polymers for their ability to inhibit crystallization, hypromellose acetate succinate HF grade (HPMCAS-HF; HF) was selected to prepare PTM ASDs. In vitro release studies were conducted in simulated fasted- and fed-state media. Drug crystallization in ASDs following exposure to dissolution media was evaluated by powder X-ray diffraction, scanning electron microscopy, and polarized light microscopy. In vivo oral pharmacokinetic evaluation was conducted in male cynomolgus monkeys (*n* = 4) given 30 mg PTM under both fasted and fed conditions in a crossover design. Three HPMCAS-based ASDs of PTM were selected for fasted-state animal studies based on their in vitro release performance. Enhanced bioavailability was observed for each of these formulations relative to the reference product that contained crystalline drug. The 20% drug loading PTM-HF ASD gave the best performance in the fasted state, with subsequent dosing in the fed state. Interestingly, while food improved drug absorption of the crystalline reference product, the exposure of the ASD formulation was negatively impacted. The failure of the HPMCAS-HF ASD to enhance absorption in the fed state was hypothesized to result from poor release in the reduced pH intestinal environment resulting from the fed state. In vitro experiments confirmed a reduced release rate under lower pH conditions, which was attributed to reduced polymer solubility and an enhanced crystallization tendency of the drug. These findings emphasize the limitations of in vitro assessment of ASD performance using standardized media conditions. Future studies are needed for improved understanding of food effects on ASD release and how this variability can be captured by in vitro testing methodologies for better prediction of in vivo outcomes, in particular for ASDs formulated with enteric polymers.

## 1 Introduction

Pretomanid (PTM), also known as PA-824, is a nitroimidazooxazine drug, which was approved by the United States Food and Drug Administration in 2019^1^ as a part of a combination regimen with bedaquiline and linezolid for the treatment of adults with pulmonary extensively drug-resistant, treatment-intolerant, or nonresponsive multidrug-resistant tuberculosis (MDR-TB).^2^ The indication was recently updated for use as part of a combination regimen with bedaquiline and linezolid for the treatment of adults with pulmonary TB that is resistant to isoniazid, rifamycins, fluoroquinolone, and second-line injectable antibacterial drugs or for adults with pulmonary TB resistant to isoniazid and rifampin, who are treatment-intolerant or nonresponsive to standard therapy.^3^ In May 2022, the World Health Organization recommended programmatic use of PTM in multidrug- and rifampicin-resistant TB.^4^

PTM is reported to be a poorly water-soluble compound with water solubility of 10–20 μg/mL^5,6^ and a log *P* of 2.75.^6,7^ Studies in humans indicate that the drug is moderately rapidly absorbed in both the fed and fasted states.^8^ The absolute oral bioavailability has not been determined in humans. In preclinical studies, the mean oral bioavailability was 19% in monkeys for a PTM suspension in 0.5% sodium carboxymethyl cellulose and 52% in rats for PTM with hydroxypropyl-β-cyclodextrin.^9^ The presence of food appears to increase the overall solubility and dissolution rate of PTM from tablets following oral delivery and thereby enhances drug absorption, especially at a high dose.^8^ Administration of 200 mg PTM with a high-calorie, high-fat meal led to an increase in the maximum concentration (*C*_max_) and the area under the curve (AUC_0-inf_) by 76 and 88%, respectively;^8^ thus, it is recommended that PTM is taken with food.^9^ However, absolute bioavailability remains low in both the fed and fasted states, suggesting that solubility-enhancing formulation strategies may be useful to improve the absorption extent.^2,8,9^

There have been only a few studies on developing solubility-enhancing formulations of PTM. Xia and co-workers used drug-impregnated mesoporous silica particles for improvement of PTM solubility.^100,11^ Drug was entrapped in the silica pores as the amorphous form at a drug loading of 28% w/w. This system exhibited higher drug release in phosphate buffer (PB) (pH 7.4) when compared to the crystalline drug. In another study, porous particles of PTM were formulated by spray drying with l-leucine and phospholipid 1,2-dipalmitoyl-*sn*-glycero-3-phosphocholine, producing a drug powder formulation for inhalation.^10,11^ The porous particles were micron-sized, showed long-term physical stability at room temperature, and resulted in a detectable drug concentration following inhalation in guinea pigs for up to 24 h.^10^ It was further noted that aerosol administration of the powder formulation exhibited much lower AUC than powder intratracheal insufflation and resulted in a lower systemic exposure (6.7–13.5%).^11^ However, treatment with an oral drug suspension showed a more significant decrease in bacterial burden in the lungs of an animal model, consistent with a higher dose relative to that achieved via inhalation.^11^ Therefore, oral dosing strategies with improved bioavailability remain of interest.

Amorphous solid dispersion (ASD) is a widely employed formulation approach for poorly soluble drugs, as evidenced by an increase in the number of commercial products utilizing this enabling strategy.^12,13^ ASD, where the drug is mixed at a molecular level with a polymer, releases drug under nonsink conditions to generate supersaturated solutions and improve release rate and in vivo absorption relative to crystalline drug.^14−19^ However, ASDs are metastable or unstable systems and tend to crystallize either in the solid matrix or from the supersaturated solution generated upon dissolution. Many polymers have crystallization inhibition properties and can be used to increase the physical stability of ASDs as well as supersaturated solutions.^19−21^ However, the diversity of polymers used in commercial ASD dosage forms is low, with examples including neutral polymers such as polyvinylpyrrolidone/co-vinyl acetate (PVPVA) and hydroxypropyl methylcellulose, or weakly acidic polymers such as hydroxypropyl methylcellulose acetate succinate and hydroxypropyl methylcellulose phthalate.^12,22^

As discussed above, drug crystallization is a primary failure mechanism for ASD formulations. The driving force for crystal nucleation and growth from solutions is the excess chemical potential of the solute relative to the saturated solution, typically described in terms of the supersaturation (*S*) using the following approximation
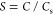
1where *C* is the solution concentration and *C*_s_ is the concentration of a saturated solution. The upper limit of supersaturation is given by the amorphous solubility; above this concentration, the solution splits into two phases: a drug-rich and a drug-lean phase. The persistence of the supersaturation generated by an ASD depends on a number of factors. These include the degree of supersaturation, the inherent crystallization tendency of the drug (from both the hydrated ASD matrix and the supersaturated solution phase), and the effectiveness of the polymer as a crystal nucleation/growth inhibitor. In terms of drug inherent crystallization tendency, one important predictor is the compound molecular weight (MW).^23^ Higher MW drugs tend to be slower crystallizers relative to their lower MW counterparts, which undergo rapid crystallization from both the amorphous and solution states.^23−25^ When assessing the performance of an ASD formulation, drug crystallization is an important consideration; if the drug crystallizes rapidly either in the ASD matrix or immediately after a supersaturated solution is produced following release, in vivo bioavailability enhancement is likely to be reduced.

The goal of this study was to evaluate the supersaturation extent and duration potential for PTM and identify effective polymeric crystallization inhibitors. A further goal was to use this insight to develop ASD formulations optimized for release and supersaturation longevity based on in vitro release testing and to evaluate a subset of these formulations in vivo. It was hypothesized that PTM would be a fast crystallizer based on its relatively low MW of 359.26 g/mol, and therefore, only a limited number of polymers would be effective crystallization inhibitors. The amorphous solubility of PTM was measured, and the induction time in the presence and absence of polymers was determined. From these results, ASD formulations were selected and ASDs were prepared by rotary evaporation and evaluated for release using one- and two-stage dissolution tests mimicking fasted- and fed-state environments. Selected formulations were prepared by spray drying and dosed to cynomolgus monkeys in the fasted and fed states using the commercial formulation, which contains crystalline PTM, as the reference product.

## 2 Experimental Section

### 2.1 Materials

PA-824 tablets (lot no. ET15057) and PA-824 drug substance (lot no. AFBH000386/2166) were manufactured by Dr. Reddy’s Labs (Telangana, India) and were supplied by TB Alliance (New York, NY). Hydroxypropyl methylcellulose phthalate (HPMCP, HP-50 and HP-55 grades), hydroxypropyl methylcellulose acetate succinate (HPMCAS, -LF, -MF and -HF grades), and hydroxypropyl methylcellulose (HPMC, substitution type 2910, grade 603) were from Shin-Etsu Chemical Co., Ltd. (Tokyo, Japan). Methacrylic acid–methyl methacrylate copolymer (Eudragit L 100) was provided by Evonik (Darmstadt, Germany). Simulated intestinal fluid powders (FaSSIF/FaSSGF) and FeSSIF V2 were purchased from Biorelevant (London, UK). Cellulose acetate phthalate (CAP), tris base, 2-dimethylaminoethanol (DMEA), meglumine, triethylamine, triethanolamine, ammediol, *N*-methyldiethanolamine (MDEA), proline sodium, and sodium lauryl sulfate (SLS) were procured from Sigma-Aldrich (St. Louis, MO).

Vitamin E d-α-tocopheryl polyethylene glycol succinate (Kolliphor TPGS) was sourced from BASF (Ludwigshafen, Germany). Sodium starch glycolate (SSG) was purchased from JRS Pharma (Rosenberg, Germany). Croscarmellose sodium (Ac-Di-Sol) and microcrystalline cellulose (MCC PH 101) were obtained from FMC Biopolymer (Newark, DE). Sodium chloride, potassium chloride, potassium phosphate monobasic, maleic acid, sodium hydroxide, sodium phosphate monobasic monohydrate, hydrochloric acid, dimethyl sulfoxide, methanol (MeOH), dichloromethane, and acetonitrile were supplied by Fisher Scientific (Pittsburgh, PA).

### 2.2 Drug Solubility Measurement

The equilibrium solubility of PTM at 37 °C was measured in aqueous media, including HCl solution (pH 1.6), phosphate buffer (PB, 50 mM, pH 3.0, 5.0, and 6.5), and maleate buffer (55 mM, pH 5.8). PTM solubility in fasted- and fed-simulated media (FaSSIF V1 and FeSSIF V2) was also evaluated. An excess of crystalline PTM was added to the test medium, and the samples were stirred at 300 rpm for 48 h. The supernatant was collected by ultracentrifugation at 35,000 rpm and 37 °C for 30 min using an Optima L-100 XP ultracentrifuge (SW 41Ti rotor) (Beckman Coulter, Inc., Brea, CA). After appropriate dilution in MeOH, the samples were assayed using an Agilent high-performance liquid chromatography (HPLC) 1260 system with a C18 column (Zorbax Eclipse Plus, 4.6 × 250 mm, 5 μm) (Agilent Technologies, Santa Clara, CA). The HPLC conditions included an injection volume of 20 μL, a mobile phase of acetonitrile and water (75:25 by volume), a flow rate of 1.5 mL/min, and UV detection at 335 nm.

The amorphous solubility in different media of interest was evaluated by the UV-extinction method.^26^ PTM was introduced gradually into the medium by adding a methanolic stock solution of drug (50 mg/mL) at a rate of 100 μL/min using a syringe pump (Harvard Apparatus, Holliston, MA). When the drug concentration exceeded the amorphous solubility, liquid–liquid phase separation (LLPS) occurred, generating scattering species that elevated the baseline of the UV/vis spectrum at 400 nm. A plot of added concentration versus extinction at 400 nm was used to determine amorphous solubility as the concentration where the extinction increases rapidly.

### 2.3 Drug Nucleation Induction Time

Drug induction time was measured using a SI Photonics UV/vis spectrometer (Tucson, Arizona), coupled with a 10 mm probe as described previously.^27^ The increase in scattering resulting from the nucleation and growth of crystals was monitored at 30 s intervals by measuring the extinction at a nonabsorbing wavelength (440 nm). The time until crystals were detected was evaluated at 37 °C at a concentration corresponding to the amorphous solubility of the drug in tested medium.

For screening the effectiveness of drug crystallization inhibition, various polymers (100 μg/mL) were pre-dissolved in PB (pH 6.5). PTM was added at a concentration of 80 μg/mL, and the nucleation induction time was measured. For HPMCAS-HF, the induction time of PTM was also determined in FaSSIF V1 and PB (pH 6.5) at drug and polymer concentrations of 160 μg/mL and 1 mg/mL, respectively.

### 2.4 Preparation of Amorphous Solid Dispersions

ASDs were prepared by solvent evaporation using a Buchi Rotavapor-R (Newcastle, DE) or a Buchi B-290 spray dryer (Newcastle, DE). ASDs were prepared by rotary evaporation for screening experiments, while formulations for animal studies were made by spray drying. Drug, polymer (HPMCAS-HF), and other additives were dissolved in a mixture of dichloromethane—methanol (1:1 v/v) at a 10% w/v solid content. For certain ASDs, counterion (i.e., base) was added at a 1:1 molar ratio to polymer based on the number of acidic functionalities in the polymer.^28^ Other components were added as a mass ratio. For rotary evaporation, solvents were removed at 45 °C under vacuum, followed by storage overnight under vacuum at room temperature. ASDs were then collected, cryo-milled, and sieved to obtain the desired particle size fraction of 106–250 μm. For animal studies, ASDs were spray-dried with an inlet temperature of 80 °C, a nitrogen gas flow rate of 700 L/h, aspirator of 95% (or 35 m^3^/h), and a feed rate of 6 mL/min, followed by storage overnight under vacuum to remove residual solvents.

### 2.5 Evaluation of ASD Physicochemical Properties

Crystallinity of ASDs was evaluated by powder X-ray diffraction (PXRD) and polarized light microscopy (PLM). For PXRD measurements, the samples were placed on glass sample holders and analyzed using a Rigaku Smartlab diffractometer (Rigaku Americas, The Woodlands, TX) equipped with a Cu   Kα radiation source and a D/tex ultradetector. Powder patterns were collected over the range of 4–40° 2θ at a scanning speed of 4° per min and a 0.02° step size, with the voltage and current set to 40 kV and 44 mA, respectively. Drug crystallization was evaluated using PLM with a Nikon Eclipse E600 POL microscope (20× objective) with an attached Nikon DS-Ri2 camera (Melville, NY).

Drug crystallization also was detected by scanning electron microscopy (SEM) using a Nova nanoSEM (FEI Company, Hillsboro, OR). A thin layer of ASD particles was added onto an aluminum stub using double-sided sticky carbon tape. Then, a thin film of platinum was applied to the samples using a sputter coater (Cressington Sputter Coater, Watford, UK) with 60 s exposure. SEM images were obtained using an Everhart-Thornley detector at a spot size of 3 nm, beam energy of 5 kV, and working distance of approximately 5 mm.

The glass transition temperature (*T*_g_) of ASD samples was measured by a TA Q2000 differential scanning calorimeter (DSC) equipped with an RCS90 refrigeration unit (TA Instruments, New Castle, DE). The temperature calibration was performed using indium and tin, while enthalpy calibration was conducted with indium. The samples (5–10 mg) were added to aluminum pans with a pinhole lid (Tzero pan, TA Instrument, DE). The sample was equilibrated at −20 °C and heated from −20 to 210 °C at 5 °C/min then cooled back down to −20 °C at 10 °C/min under a nitrogen flow of 50 mL/min. The heating and cooling cycle was repeated three times to remove residual solvent and thermal history, and the last cycle was used for analysis.

Drug impurities in ASDs were examined by ^19^F nuclear magnetic resonance (NMR) spectroscopy. Samples were dissolved in dimethyl sulfoxide-d6 (Cambridge Isotope Laboratories, Inc., Andover, MA) at a concentration of 20 mg/mL. All NMR spectra were acquired on a Bruker DRX 500 MHz spectrometer (Karlsruhe, Germany) equipped with a BBFO z-gradient probe operating at room temperature. For ^19^F NMR spectroscopy, ^1^H was decoupled during acquisition; the spectral sweep width was 50 ppm, the acquisition time was 1.4 s, and the number of scans was 64.

### 2.6 Release Testing

Dissolution studies were conducted in triplicate under single-stage (FaSSIF V1, pH 6.5, or FeSSIF V2, pH 5.0–6.0) or two-stage pH-shift conditions (FaSSGF, pH 1.6, for 1 h followed by FaSSIF V1, pH 6.5, for another 1 h) using a USP apparatus II Hanson Vision G2 Classic 6 dissolution system (Teledyne Hanson Research, Chatsworth, CA). For screening ASD formulations, an amount of ASD powder equivalent to 10 mg drug was added to the dissolution medium at 37 °C with a stirring rate of 150 rpm. The dissolution medium volume was 50 mL for single-stage dissolution. For pH-shift experiments, the samples were immersed in 45 mL FaSSGF, pH 1.6, for 1 h, followed by the addition of 5 mL of 10× concentrated FaSSIF solution (in 0.57 M PB, pH 7.3^29^) to achieve 50 mL FaSSIF, pH 6.5. The maximum theoretical concentration for complete drug release was 200 μg/mL.

For tablets/capsules containing 30 mg PTM prepared for in vivo study, the dissolution medium volume was adjusted to 150 mL to achieve a similar maximum drug concentration. Dissolution tests were conducted at a stirring rate of 75 or 150 rpm. An in situ Rainbow fiber optic ultraviolet spectrometer with a 10 mm pathlength fiber optic probe (Pion, Billerica, MA, USA) was used to monitor the drug concentration over time. Second derivative analysis was applied to correct the spectral baseline and a calibration curve of AUC of the range 390–410 nm versus concentration was generated to calculate the released drug concentration.

### 2.7 Preparation of Formulations for In Vivo Study

For the in vivo pharmacokinetics (PK) study in monkeys, all formulations were prepared at a dose of 30 mg (Table 1). The tablet and capsule sizes used were suitable for administration to monkeys. Reference PA-824 tablets were crushed into powder, and 120 mg of the resultant granules (equivalent to 30 mg PA-824) were filled into size 0 HPMC capsule shells (Ezee Lock, Clear) (AlfaCaps LLC, New York, NY). Spray-dried ASD (150 mg) was mixed with excipients, including Ac-Di-Sol (30 mg), SSG (30 mg), and MCC (90 mg), and compressed using round die and punch with a target tablet weight of 300 mg and 3–4 kp hardness. The tablets were 11 mm in diameter and 4.3 mm in thickness.

**Table 1:** Experimental Design of In Vivo PK Study in Monkeys

formulation	fed/fasted status	batch no
capsule of crushed PTM tablet (reference)	fasted	PA-824-30MG-002
	fed	
tablet of PA-824:HPMCAS-HF 20:80 ASD (PTM-HF ASD)	fasted	PA-824-HF-SD-002
	fed	
tablet of PA-824:HPMCAS-HF-Tris 20:80 ASD (PTM-HF-Tris ASD)	fasted	PA-824-HF-Tris-SD-002
tablet of PA-824:HPMCAS-HF-TPGS 20:80 ASD (PTM-HF-TPGS ASD)	fasted	PA-824-HF-TPGS-SD-002

The tablets or capsules were packaged and stored in 2 oz high-density polyethylene bottles with desiccant at ambient room temperature after preparation.

### 2.8 In Vivo PK Study in Monkeys

#### 2.8.1 Animals

The in vivo part of the study was conducted at WuXi AppTec (Suzhou) Co, Ltd. in Suzhou, China. All animal experiments were conducted in compliance with the Animal Welfare Act^30^ and the Guide for the Care and Use of Laboratory Animals.^31^ Approval from the Institutional Animal Care and Use Committees at WuXi AppTec was obtained before conducting the in vivo PK study.

Cynomolgus monkeys were selected as the nonrodent species for PTM formulation testing due to rapid clearance and poor absorption in dogs, resulting in very low systemic exposures regardless of the formulation and route of administration tested.^32^ Cynomolgus macaques (*Macaca fascicularis*) were obtained from GuangDong Blooming-Spring Biological Technology Development Co., Ltd (Guangdong, China) and were confirmed to be healthy by WuXi Veterinarians (Suzhou, China) before testing. Body weights of the monkeys were between 2.75 and 3.64 kg during the study. Monkeys were housed individually in stainless-steel cages under standard conditions of temperature, humidity, ventilation, and illumination. Fresh drinking water (reverse osmosis) was provided ad libitum, and the animals were fed twice daily on nondosing days (approximately 120 g/day). Feeding schedules for dosing days are described in the experimental design below.

#### 2.8.2 Experimental Design

Four non-naive, male cynomolgus monkeys were orally administered PTM (30 mg/animal) formulations as a single capsule or tablet in a six-phase crossover design with at least a 7 day washout period between each dosing formulation/condition. Monkeys were administered formulations as shown in Table 1.

On dosing days for the fed state, animals were provided food approximately 30 min prior to dosing and food consumption was measured (animals consumed between 44 and 73 g of food confirming the fed state). For the fasted state, food was withheld overnight and returned the next day 4 h post dosing. Blood samples were collected prior to dosing and at 0.25, 0.5, 1, 2, 4, 8, 24, 28, 32, and 48 h post dose into K_3_EDTA tubes and processed into plasma for PK evaluation. Concentrations of PTM in plasma samples were determined by a liquid chromatography tandem mass spectrometry (LC–MS/MS) method.

#### 2.8.3 Bioanalytic Method

An LC–MS/MS method was developed for the quantitation of PTM in monkey plasma. PTM and internal standards (labetol, dexamethasone, tolbutamide, verapamil, glyburide, and celocoxib in acetonitrile) were extracted from 20 μL monkey plasma by protein precipitation, separated by an Acquity UPLC HSS T3 column (1.8 μm, 2.1 × 50 mm) (Waters, Ireland) with a run time of 1 min. Electrospray ionization was performed using Sciex Triple Quad 6500 Plus or API 4000 (Sciex, MA, USA), which was operated in positive ion multiple reaction monitoring mode (PTM *mz* transitions 360.20/175.10) with a dynamic range of 1–3000 ng/mL. Quality control samples were prepared in monkey plasma at five concentrations (3, 40, 800, 2400, and/or 4000 ng/mL) to monitor the assay performance.

#### 2.8.4 Pharmacokinteic Data Analysis

The PK parameters for PTM were derived from the individual plasma concentration time profiles based on noncompartmental analysis using Phoenix WinNonlin v6.3 (Pharsight, Mountain View, CA, USA) with linear up/log down trapezoidal rule. A nominal time was used for all PK calculations. The mean area under the concentration curve from time zero to infinity (AUC_0-inf_) values normalized to the actual dose (expressed as mg/kg) were used to calculate the relative bioavailability compared with the capsule of crushed PA-824 tablet in the fasted state. For the calculation of PK parameters, plasma PTM concentrations below the lower limit of quantitation were set to zero prior to the first quantifiable concentration and as missing thereafter.

### 2.9 Statistical Analysis

The statistical significance was calculated using one-way analysis of variance (ANOVA), Duncan’s method. The level of statistical significance between groups was considered as significant if *p* < 0.05 (*) or very significant if *p* < 0.01 (**) or *p* < 0.001 (***). For plasma PTM concentration, the value of zero was used for the mean and standard deviation calculations, if ⩽50% of samples had measurable concentrations.

## 3 Results

### 3.1 Evaluation of Drug Physicochemical Properties

PTM is a 4-nitroimidazole derivative with poor aqueous solubility and a log *P* of 2.75.^7^ The experimental equilibrium solubility of the drug at 37 °C ranged between 15 and 18 μg/mL in various buffer solutions and was independent of pH, in line with the PTM chemical structure (Table 2). The amorphous solubility increased relative to the crystalline form, with an amorphous/crystalline solubility (A/C) ratio of 8–10. PTM amorphous solubility in PB (pH 6.5) was 166.1 ± 6.2 μg/mL and was increased by the presence of surfactants (SLS or TPGS at a concentration of ∼200 μg/mL, Figure S1). The equilibrium solubility was increased by a factor of ∼3 in FaSSIF V1 and a factor of ∼6 in FeSSIF V2.

**Table 2:** Drug Solubility and Induction Times in Different Media^a^^b^

medium	crystalline solubility (μg/mL)	amorphous solubility (μg/mL)	A/C ratio	induction time (min) at 160 μg/mL
HCl solution (pH 1.6)	15.2 ± 1.4	141.0 ± 3.7	9.3	1.6 ± 0.2
phosphate buffer (pH 3.0)	17.8 ± 0.3	143.3 ± 2.4	8.1	1.3 ± 0.1
phosphate buffer (pH 5.0)	16.8 ± 0.1	159.6 ± 5.7	9.5	1.6 ± 0.2
phosphate buffer (pH 6.5)	16.2 ± 1.4	166.1 ± 6.2	10.2	1.4 ± 0.2
maleate buffer (pH 5.8)	17.0 ± 0.3	N/A	N/A	N/A
FaSSIF V1 (pH 6.5)	53.8 ± 4.0	308 ± 12	6.0	0.9 ± 0.3
FeSSIF V2 (pH 5.8)	108.1 ± 0.1	N/A	N/A	N/A

a Mean values ± standard deviations, *n* = 3.

b A/C ratio: amorphous/crystalline solubility ratio. N/A: not applicable.

PTM had a *T*_g_ of 10 °C and a high tendency to crystallize from both the amorphous material and supersaturated solution. Glassy PTM could only be obtained by quenching the melt in liquid nitrogen; however, poor glass stability was observed with rapid crystallization upon heating to just above the *T*_g_, followed by melting of the resultant crystals at 147.3 °C (Figure S2A). Drug crystallization of initially glassy PTM was also visually observed after storing the material at room temperature for a few minutes (Figure S2B). Further, drug crystallization occurred rapidly in solution, with a nucleation induction time of less than 2 min (Table 2).

**Figure 1:**
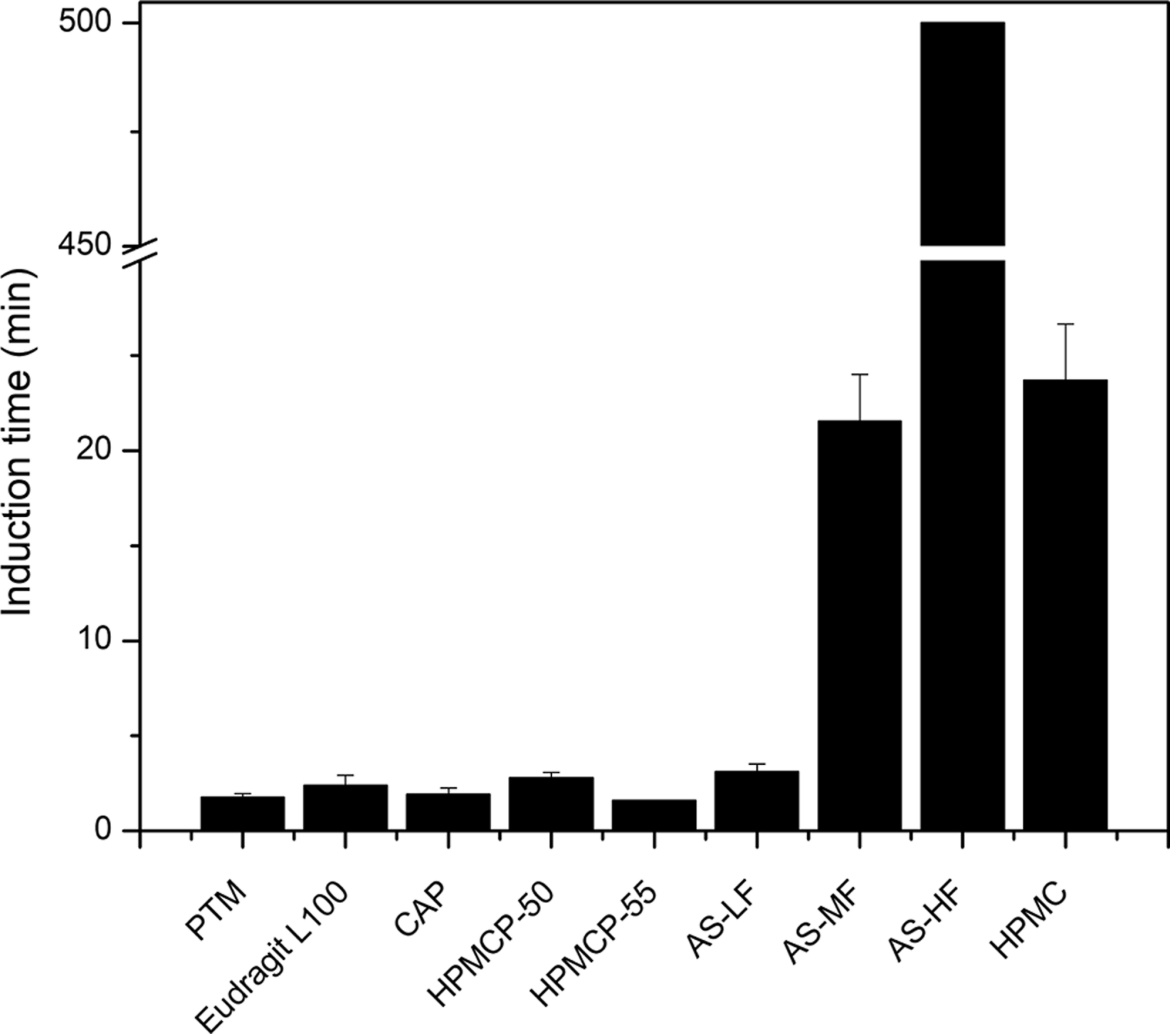
Induction time of PTM at a drug concentration of 80 μg/mL in PB (pH 6.5) with pre-dissolved polymer at a concentration of 100 μg/mL. Error bars represent standard deviation, *n* = 3.

Polymers have been used widely as drug crystallization inhibitors.^21,33^ Several polymers were screened to test their effectiveness in inhibiting the nucleation and crystal growth of PTM. The induction time of the drug at a concentration of 80 μg/mL (supersaturation ratio of 5) was measured in PB (pH 6.5) in the presence of different polymers pre-dissolved at a concentration of 100 μg/mL. Most of the enteric polymers evaluated, including Eudragit L-100, CAP, HPMCP (P-50 or P-55 grade), and HPMCAS-LF, were found to be ineffective at inhibiting drug crystallization with very short induction times of 2–5 min being observed (Figure 1). However, the more hydrophobic HPMCAS grades were more effective in inhibiting the crystallization of PTM. In the presence of HPMCAS-MF, crystallization was delayed to ∼22 min, and a similar induction time was observed for the neutral polymer, HPMC. Furthermore, no crystallization was observed in the presence of HPMCAS-HF for up to 1000 min.

The supersaturation ratio was increased to approximately 10 (PTM concentration of 160 μg/mL) and HPMCAS-HF was re-evaluated to determine its effectiveness as a crystallization inhibitor when there is a higher thermodynamic driving force for nucleation. This PTM concentration approximates the amorphous solubility (Table 2). For an HPMCAS-HF concentration of 1 mg/mL, evidence of drug crystallization was observed after approximately 44 and 77 min, in PB (pH 6.5) and FaSSIF, respectively (Table S1). However, when the drug concentration exceeded the amorphous solubility, HPMCAS-HF was not able to effectively prevent nucleation in the presence of drug-rich droplets, and crystallization occurred within a few minutes (Figure S3). Based on its effectiveness as a crystallization inhibitor, HPMCAS-HF was selected for further exploration for PTM ASD formulations.

### 3.2 PTM Release from ASDs

Release of PTM from HPMCAS-HF-based ASDs was evaluated with powders prepared by rotary evaporation in order to screen various formulations. Given that HF is the most hydrophobic grade of HPMCAS (due to the high ratio of acetyl to succinoyl substituents) and requires a higher pH to trigger polymer dissolution compared to other HPMCAS grades,^34,35^ several strategies were investigated in an attempt to enhance release while optimizing crystallization inhibition. These strategies included combining different HPMCAS grades, addition of surfactant, or adjusting the microenvironment in the ASD by adding a basic compound.

**Figure 2:**
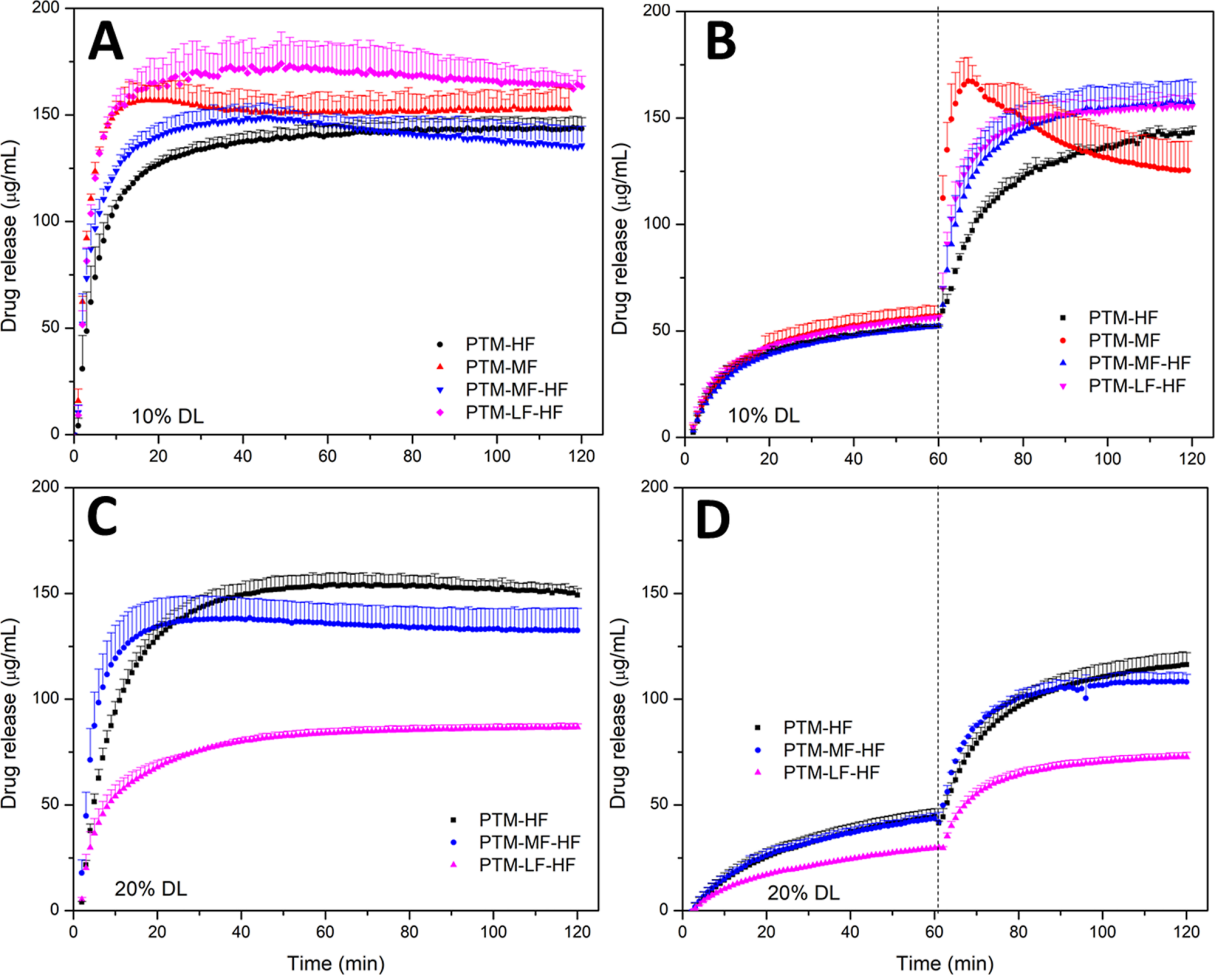
Release profiles for PTM-HPMCAS ASDs with different HPMCAS grades at (A,B) 10 wt % and (C,D) 20 wt % DL in (A,C) FaSSIF V1 and (B,D) a pH-shift experiment where the dashed line indicates a shift from FaSSGF to FaSSIF. The maximum theoretical drug release was 200 μg/mL. Error bars represent standard deviation, *n* = 3.

PTM ASDs with either a single HPMCAS grade or a combination of grades were prepared at a 10 or 20 wt % drug loading (DL). At 10 wt % DL, the PTM-HF ASD exhibited a slower and slightly lower extent of drug release in FaSSIF V1 compared to the corresponding ASD prepared with HPMCAS-MF (Figure 2A). However, for the pH-shift two-stage release test, a decrease in solution concentration was observed for the PTM-MF ASD (Figure 2B) upon transfer from simulated gastric fluid to FaSSIF V1, consistent with crystallization. Combining HPMCAS-HF and either the -MF or -LF grade (50:50 weight ratio of the polymers) led to improved release profiles at a 10 wt % DL (Figure 2A,B). Unfortunately, for the 20 wt % DL, a reduced extent of PTM release was observed for the polymer blends, especially for the ASD containing a combination of HPMCAS-LF and -HF (Figure 2C,D). This was likely due to the surface crystallization of PTM and the agglomeration of ASD particles that were observed following the initial dispersion of the powder in the dissolution media (Figures S4 and S5).

#### 3.2.1 Impact of Surfactant on Drug Release of PTM ASDs

Another strategy evaluated to improve release from ASDs formulated with HPMCAS-HF was surfactant addition. Surfactants have been noted to positively impact ASD performance by increasing wettability, dispersibility, and drug solubility.^17,18,36−39^ However, surfactants may also promote drug crystallization or phase separation, phenomena expected to be detrimental for drug release.^36,40^

**Figure 3:**
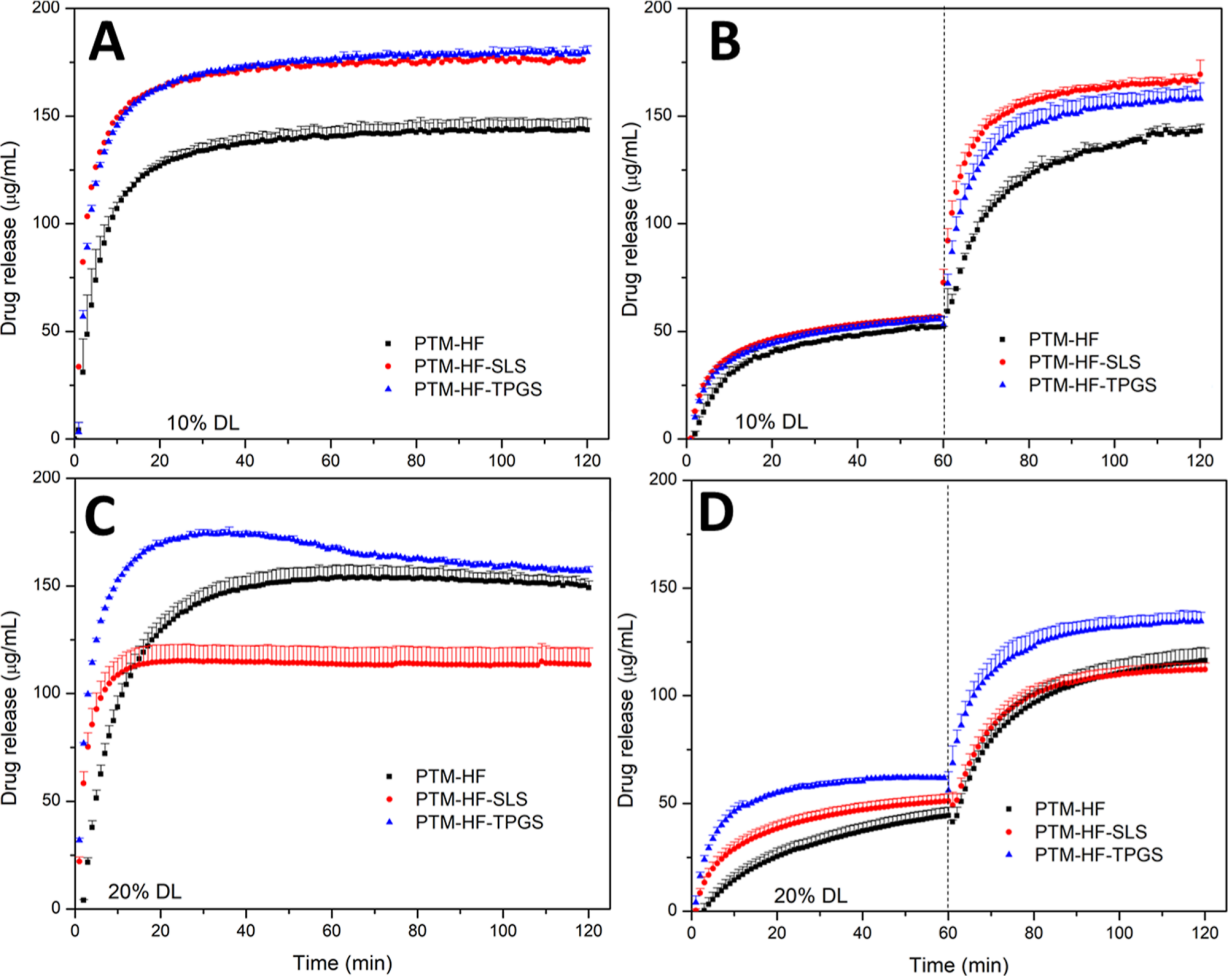
Dissolution of PTM-HF ASDs in the presence of surfactant (10% w/w versus PTM) at (A,B) 10% and (C,D) 20% DL in (A,C) FaSSIF V1 and (B,D) pH-shift experiments where the dashed line indicates a shift from FaSSGF to FaSSIF. The maximum theoretical drug release was 200 μg/mL. Error bars represent standard deviation, *n* = 3.

For PTM-HF ASDs, SLS and TPGS were added at a 10 wt % ratio relative to the drug load. At a low drug loading (10 wt %), the addition of surfactant improved PTM release for both single-stage and pH-shift release tests (Figure 3A,B). Incorporation of either SLS or TPGS resulted in drug release of nearly 90% in FaSSIF alone versus approximately 75% in the pH-shift experiment. However, when the drug loading was increased to 20%, SLS addition failed to enhance release, while TPGS incorporation still led to improvements relative to the binary ASD (Figure 3C,D).

**Figure 4:**
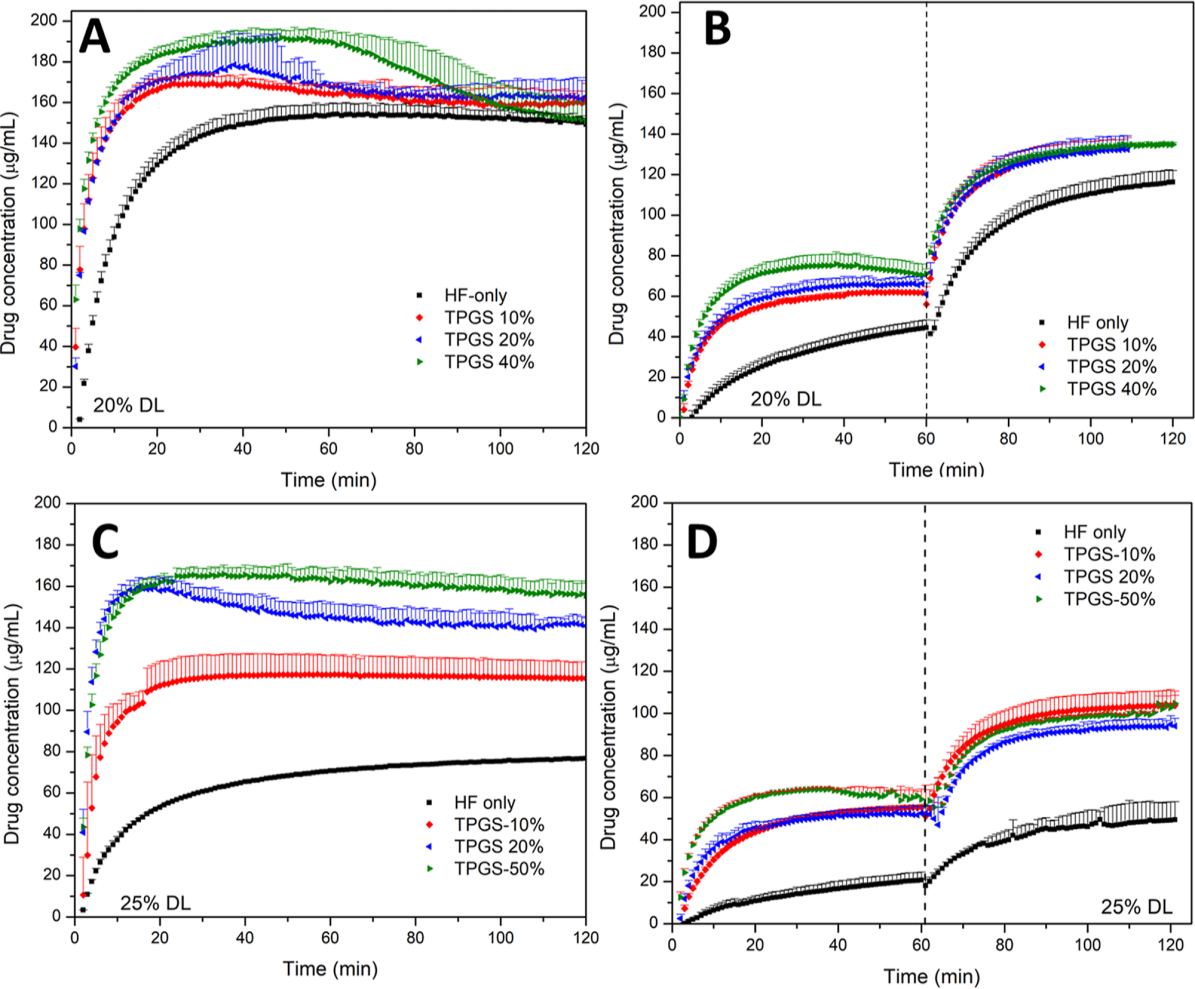
Dissolution of PTM-HF-TPGS ASDs with different TPGS/drug ratios at (A,B) 20% DL and (C,D) 25% DL in (A,C) FaSSIF V1 and (B,D) pH-shift experiment with the dashed line indicating a media shift from FaSSGF to FaSSIF V1. The maximum theoretical drug release was 200 μg/mL. Error bars represent standard deviation, *n* = 3.

The influence of TPGS on the release performance of PTM-HF ASDs was further assessed as a function of the TPGS/drug ratio at both a 20 and 25 wt % DL (Figure 4). TPGS plasticized the ASD formulation, leading to a reduction in *T*_g_ (Figure S6). Release of PTM-HF ASDs in FaSSIF dropped notably when the drug loading was increased, from about 150 μg/mL after 60 min at 20% DL to less than 70 μg/mL at a 25% DL (Figure 4A,C). The presence of TPGS led to remarkably improved release in the single-stage FaSSIF test. Addition of 20% TPGS (relative to the amount of PTM) led to improved PTM concentrations of about 180 and 150 μg/mL for 20 and 25% DL, respectively. The amount of TPGS in the ASD produced a larger impact on the release profiles (single-stage testing) for ASDs with 25 wt % DL. For pH-shift experiments, higher TPGS concentration in the ASD led to increased release of drug in FaSSGF (Figure 4B,D), but no additional improvement upon switching to the FaSSIF stage. Thus, the ASD formulation at 20% DL in the presence of 20% TPGS was selected for further experiments.

#### 3.2.2 Impact of the Incorporation of a Basic Compound in the ASD

HPMCAS-HF is a weakly acidic polymer, whereby polymer dissolution requires a critical fraction of the carboxylic acid groups to be ionized, which in turn requires a threshold pH to be reached.^41^ Adding a base to the ASDs is expected to enhance the polymer dissolution rate by altering the microenvironmental pH, and therefore, the drug release.^28^ Herein, several basic additives (primary, secondary, and tertiary amines) were added at a 1:1 polymer/base molar ratio (using the number of carboxylic acid group in HPMCAS-HF provided by the manufacturer)^35^ for ASDs with a 20 wt % DL. Various properties of the basic additives are summarized in Table S2.

**Figure 5:**
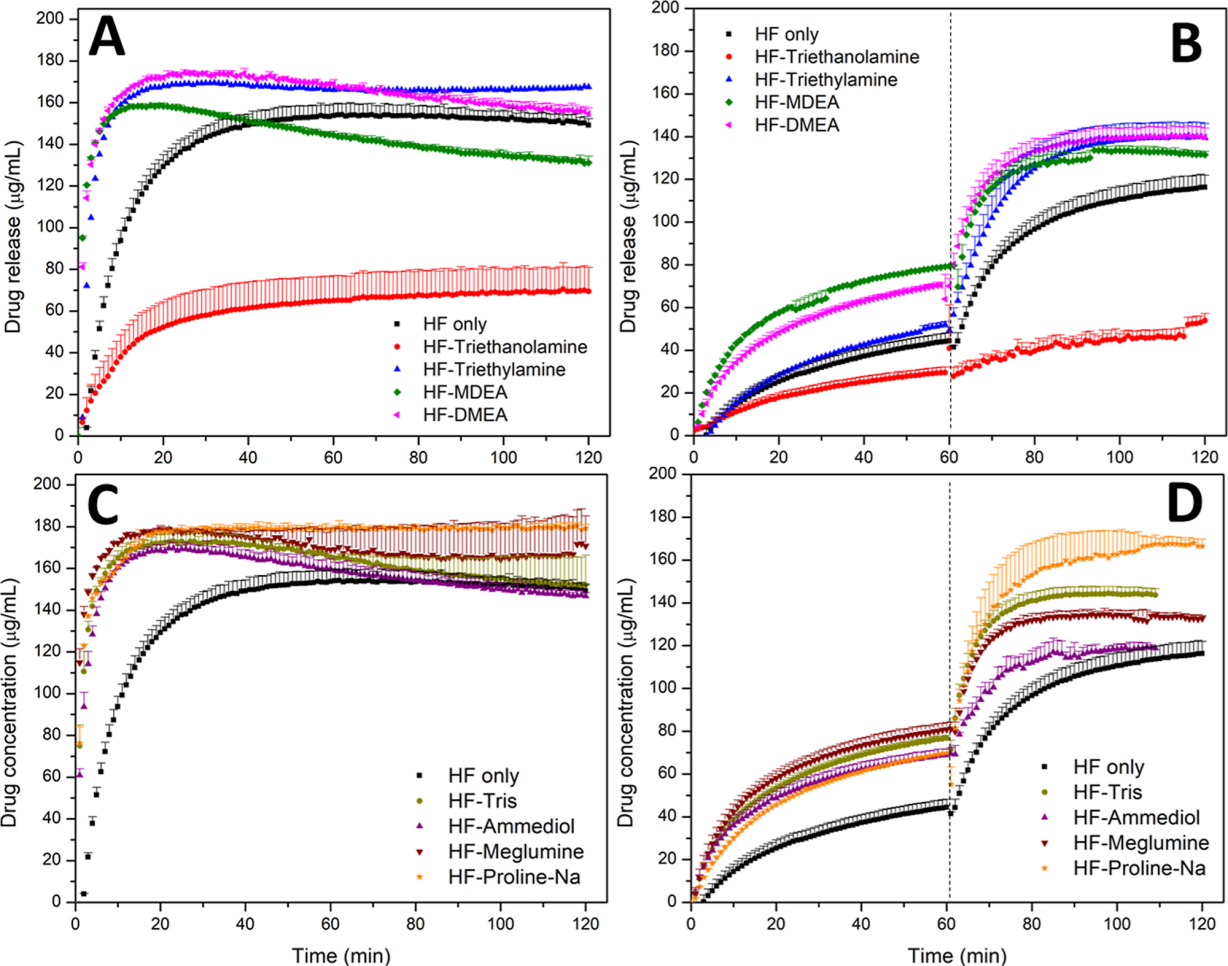
Release profiles of ASDs of PTM-HF with addition of amine compounds at a 20 wt % DL with (A,B) tertiary amine ASDs and (C,D) primary and secondary amine ASDs in (A, C) FaSSIF-V1 and (B,D) pH-shift experiment with the dashed line indicating a media shift from FaSSGF to FaSSIF. The maximum theoretical drug release was 200 μg/mL. Error bars represent standard deviation, *n* = 3.

In comparison to the binary PTM-HF ASD (black line), the addition of an amine led to more rapid and extensive drug release for single-stage dissolution, except in the case of triethanolamine, which is the base with the lowest p*K*_a_ (7.73 at 25 °C)^42^ (Figure 5A,C). Greater differentiation between the bases was observed in the two-stage dissolution testing with proline sodium and tris providing the greatest extents of release (Figure 5B,D). However, the proline sodium ASD appeared to show reduced drug stability based on shifts in drug peak positions in this system observed in ^19^F NMR spectra (Figure S7). Furthermore, some of the other bases do not have well-established in vivo safety profiles. Consequently, tris (also known as trometamol), which has been used as a cationic counterion in approved products,^43^ and thus has an established safety profile, was selected for further experiments.

### 3.3 Release Studies of Formulations Used for In Vivo Studies

For in vivo studies, selected PTM ASDs were compressed into tablets containing 30 mg of PTM. Release of ASD tablets as well as capsules containing a powder derived from the reference product were conducted under fasted/fed single-stage or fasted-state two-stage conditions.

**Figure 6:**
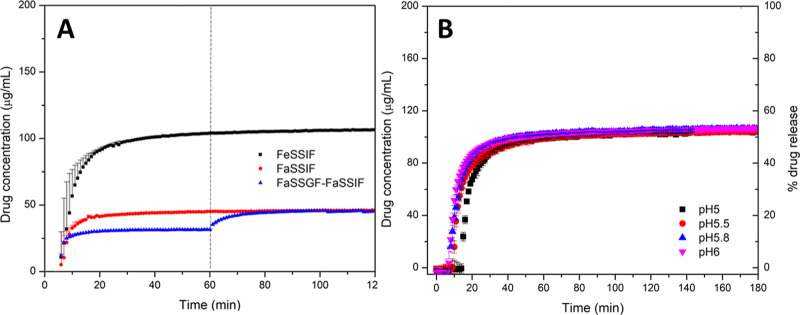
Release profiles of capsules containing a powder of the reference tablet in (A) simulated gastric fluid (pH 1.6) followed by fasted-state simulated intestinal media (FaSSIF V1, pH 6.5) with the dotted line indicating the transition point from gastric to intestinal media (blue), or single-stage release in FaSSIF V1, pH 6.5 (red), or fed-state simulated intestinal fluid (FeSSIF V2, pH 5.8) (black) with a stirring rate of 150 rpm and (B) FeSSIF V2 at different pH values with a stirring rate of 75 rpm. The maximum theoretical drug release was 200 μg/mL. Error bars represent standard deviation, *n* = 3.

To evaluate the reference product, commercial PTM tablets were crushed into a powder and filled into size 0 HPMC capsules to obtain a 30 mg dose of PTM suitable for animal studies. Release testing indicated that after a lag-time of about 5–6 min due to dissolution of the capsule shell (Figure 6A), the release profiles of the capsules were similar to those of the original tablets (Figure S8). The commercial product is an immediate release formulation of PTM in crystalline form and contains SLS.^44^ The presence of the surfactant likely increases the solubility of crystalline PTM; the drug concentration in gastric fluid was about 30 μg/mL, almost double the crystalline solubility in HCl solution, pH 1.6 (Table 2).

In FaSSIF V1, drug release increased relative to in gastric fluid, consistent with drug solubilization by biorelevant media. In fed-state media, a notable increase in the extent of PTM dissolution was observed, where a final concentration of 110 μg/mL was seen in FeSSIF V2 (pH 5.8), close to PTM crystalline solubility in this medium (Table 2). Importantly, the reference formulations showed consistent release profiles, regardless of the pH of the dissolution media or the stirring rate (Figure 6B). The same release rate and maximum drug concentration were found in FeSSIF across the pH range of 5–6 and for a stirring rate of either 75 or 150 rpm (Figure 6B).

**Figure 7:**
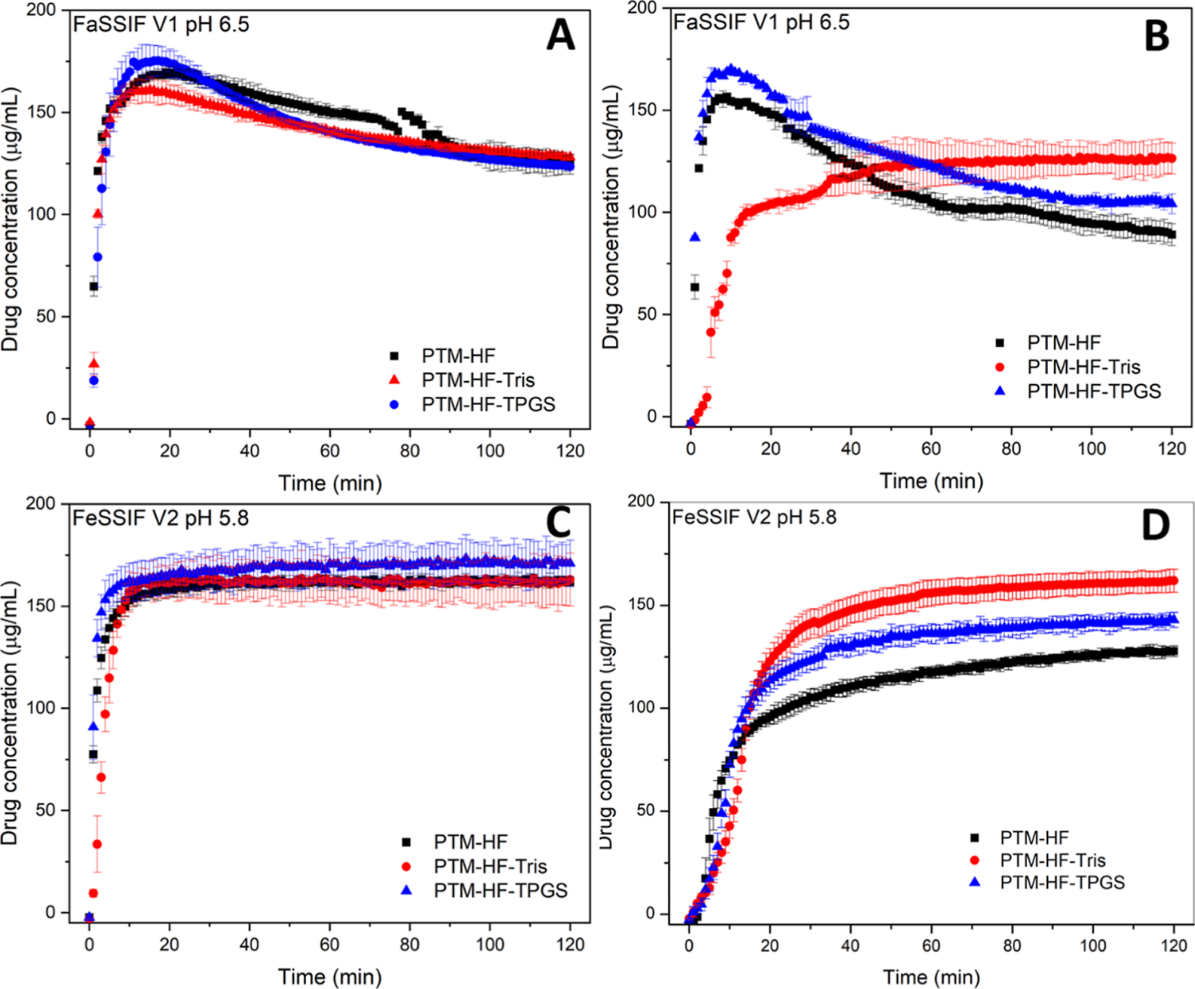
Release profiles for PTM ASD tablets at 20% DL in (A,B) fasted-state simulated media (pH 6.5) and (C,D) in fed-state simulated intestinal fluids at (A,C) 150 rpm and (B,D) 75 rpm. The maximum theoretical drug release is 200 μg/mL. Error bars represent standard deviation, *n* = 3.

**Figure 8:**
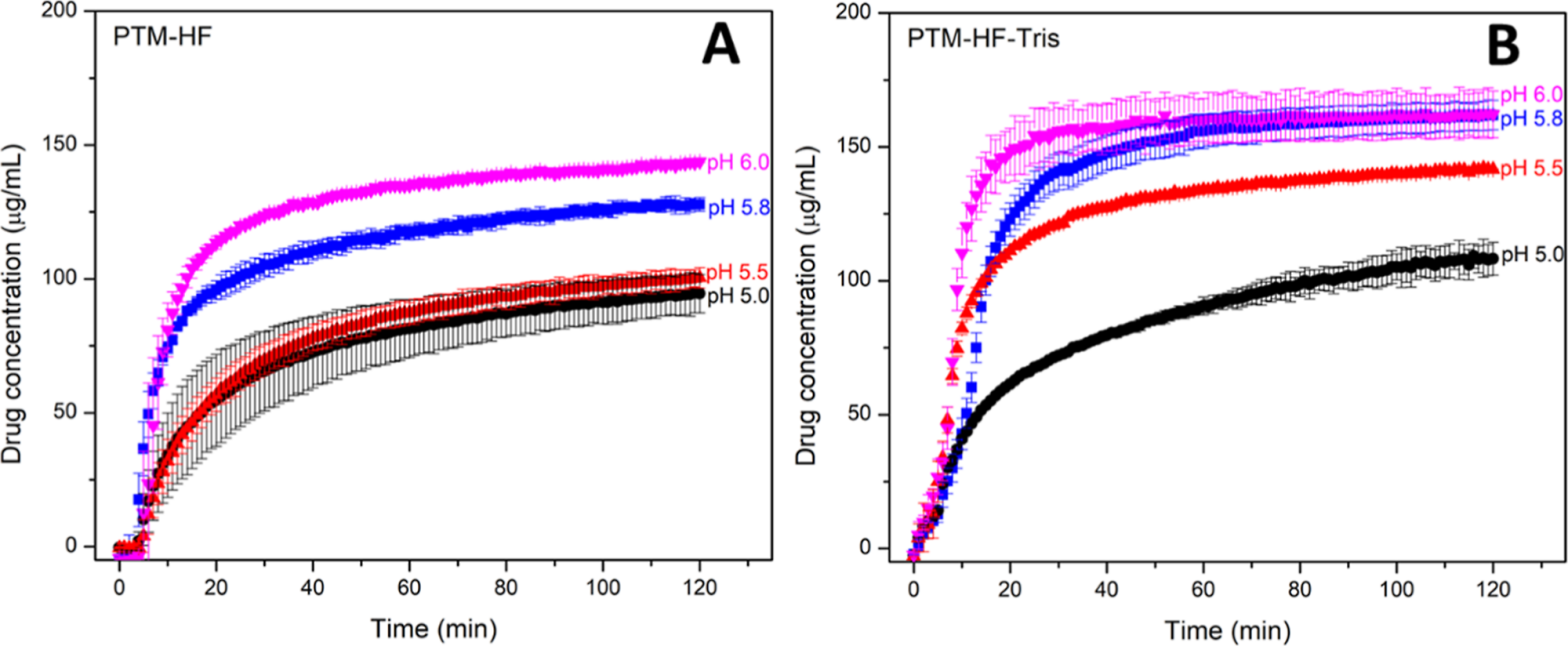
Impact of medium pH for the pH range 5.0–6.0 on drug release from (A) PTM-HF ASD tablets and (B) PTM-HF-Tris ASD tablets in FeSSIF at 75 rpm stirring speed. The maximum theoretical drug release was 200 μg/mL. Error bars represent standard deviation, *n* = 3.

**Figure 9:**
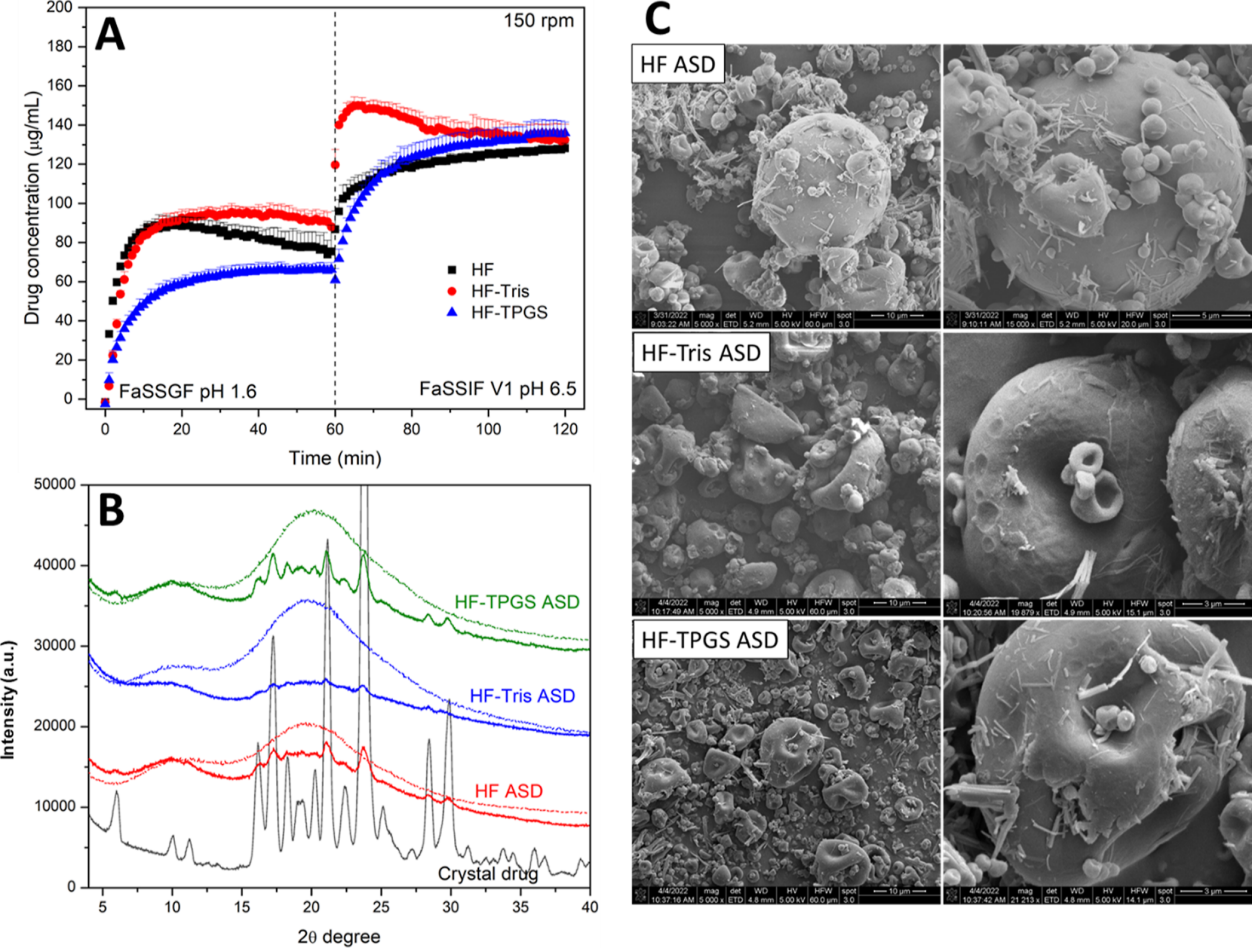
(A) Drug release from ASD tablets (20% DL) in pH-shift experiments at 150 rpm stirring speed with the dotted line indicating the transition point from gastric (FaSSGF) to intestinal (FaSSIF) media. The maximum theoretical drug release was 200 μg/mL. Error bars represent standard deviation, *n* = 3. (B) PXRD of ASDs before (dashed line) and after (solid line) acidic immersion. (C) SEM images showing the surface morphology of ASD particles after acidic immersion.

Release profiles of PTM from ASD tablets are summarized in Figures 7–9. PTM ASD tablets exhibited faster and more extensive release than the capsule containing the reference formulation. The maximum drug concentration under single-stage FaSSIF conditions was about 170 μg/mL for the PTM-HF ASD, with minor improvements seen for the ASDs containing tris or TPGS (TPGS/drug ratio of 20%) (Figure 7A). Interestingly, even though ASDs were prepared with HPMCAS-HF, which has a p*K*_a_ > 5^45^ and reported dissolution pH threshold of 6.5,^35^ PTM ASDs still showed a good drug release in FeSSIF at pH 5.8 (Figure 7C). Release from PTM ASD tablets was found to be dependent on the hydrodynamic conditions employed in the experiment. Slower mixing (i.e., reduced stirring rate) resulted in reduced drug release, in both fasted and fed simulated fluids (Figure 7B,D). Further, PTM-HF-Tris ASD tablets experienced much slower disintegration and formed larger agglomerates, as compared with the PTM-HF formulation (Figure S9).

When the pH of FeSSIF was modified to more acidic values (pH 5.0 or 5.5), the PTM-HF ASD exhibited more limited drug release, achieving a maximum concentration close to the drug crystalline solubility (∼110 μg/mL in FeSSIF, Table 2) (Figure 8A). In higher pH FeSSIF media (pH 5.8 and 6.0), the drug concentration exceeded the crystalline solubility, and a supersaturated solution was formed and maintained. The presence of a basic additive lowered the pH threshold for enhanced drug release for the PTM-HF-Tris ASD to 5.5 (Figure 8B). Matrix crystallization was noticed for ASDs of PTM dissolving in low pH simulated intestinal fluids (confirmed by XRD in Figure S10A,B and SEM images in Figure S10C,D) likely accounting for the incomplete drug release observed.

In pH-shift experiments, a greater extent of drug release in the FaSSGF compartment (pH 1.6) was observed for the ASDs (Figure 9A) as compared to the crystalline reference formulation (Figure 6A), followed by additional release in the FaSSIF stage (pH 6.5), leading to higher final drug concentrations relative to the reference formulation. However, the total release extent was reduced in two-stage dissolution (Figure 9A) relative to single-stage (Figure 7A), suggesting a deleterious change during immersion in the gastric stage, most likely due to drug crystallization. To further evaluate this, ASD particles were analyzed using PXRD and SEM imaging following immersion in gastric media for 1 h. Evidence of PTM crystallization was observed via the presence of diffraction peaks on PXRD diffractograms of each of the ASDs following gastric immersion (Figure 9B), where qualitatively, the extent of crystallinity followed the order HF-TPGS > HPMCAS > HPMCAS-Tris. SEM images revealed the presence of needle-shaped crystals on the surface of ASD particles after gastric immersion (Figure 9C).

### 3.4 Pharmacokinetics of ASD Tablet Formulations After Oral Administration to Monkeys

**Figure 10:**
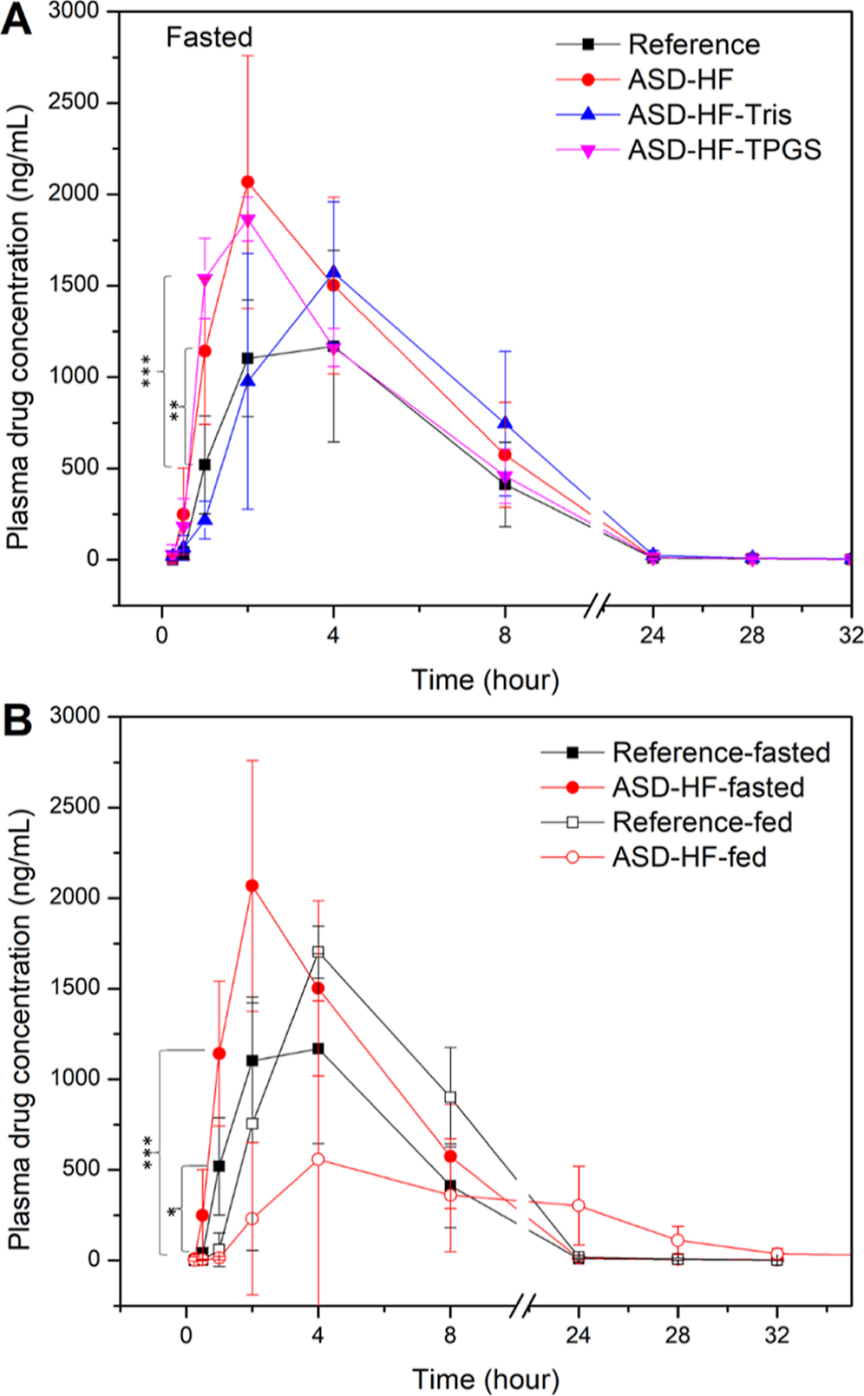
Plasma concentration of PTM in male cynomolgus monkeys following a single oral administration of PTM formulations (30 mg) (A) in fasted state and (B) the effect of food on selected formulations. Level of statistical significance at 1 h: *p < 0.05; **p < 0.01; and ***p < 0.001. Error bars represent standard deviation, *n* = 4.

The fasted-state oral absorption of three PTM ASD formulations was evaluated in cynomolgus monkeys (*n* = 4) at an oral dose of 30 mg drug, in comparison to the reference formulation (crushed commercial PA-824 tablets in HPMC capsule size 0) (Figure 10A). The ASD formulations of PTM improved the relative bioavailability in comparison to the reference product that contained crystalline drug. Although *C*_max_ values were not significantly different, PTM ASDs of HPMCAS-HF only or -HF with TPGS exhibited faster absorption with higher plasma concentration after 1 h compared with the reference formulation. Delayed release and absorption were observed for the ASD with the polymer salt (HF-Tris) whereby the *T*_max_ was 4 h, relative to 2 to 2.5 h for the other formulations (Table 3). The highest AUC was observed for the binary PTM-HF-ASD, which had a relative bioavailability of 155% compared to crystalline formulation (*p* < 0.05).

**Table 3:** Mean PK Parameters of PTM in Male Cynomolgus Monkeys Following a Single Oral Administration of Reference or ASD Formulations of PTM (30 mg) in Fasted and Fed states^a^^b^

PK parameters	reference	PTM-HF ASD	PTM-HF-Tris ASD	PTM-HF-TPGS ASD	reference	PTM-HF ASD
	fasted	fasted	fasted	fasted	fed	fed
*C*_max_ (ng/mL)	1385 (355)	2083 (673)	1573 (388)	1865 (120)	1702 (143)	765.8 (743.6)^c^^,^^d^^,^^e^^,^^f^
*T*_max_ (h)	2.50 (1.00)	2.50 (1.00)	4.00 (0.00)	2.00 (0.00)	4.00 (0.00)	19.00 (10.00)
*T*_1/2_ (h)	2.73 (0.49)	2.76 (0.50)	3.54 (1.23)	3.15 (0.43)	2.85 (0.22)	3.01 (0.51)
AUC_0-24h_ (ng.h/mL)	7805 (2742)	11751 (3483)	10870 (4307)	10161 (1246)	11575 (1379)	7142 (3037)^c^^,^^f^
AUC_0-last_ (ng.h/mL)	7836 (2776)	11802 (3514)	10996 (4451)	10221 (1299)	11650 (1415)	8315 (2474)^c^^,^^f^
AUC_0-inf_ (ng.h/mL)	7846 (2780)	11815 (3518)	11006 (4450)	10238 (1313)	11663 (1424)	8353 (2440)
MRT_0-inf_ (h)	5.57 (1.14)	5.38 (1.33)	6.82 (1.12)	5.33 (0.91)	7.19 (1.02)	16.03 (6.24)
relative bioavailability (%)		155	142	141		
food-effect relative bioavailability (%)					163	72.2

a Mean (standard deviation) values reported, *n* = 4.

b AUC: Area under the concentration–time curve; from time 0 to *x*; Inf: infinity; MRT: Mean residence time; individual animal AUC_0-inf_ values were normalized to actual dose (in mg/kg) and compared with reference fasted AUC_0-inf_ for calculating the relative bioavailability. Individual animal fed AUC_0-inf_ values were normalized to actual dose (in mg/kg) and compared with the corresponding formulations fasted AUC_0-inf_ to calculate the food-effect relative bioavailability.

c Statistically significant difference between groups: *p* < 0.001 compared with the PTM-HF ASD (fasted) group.

d *p* < 0.05 compared with the PTM-HF-Tris ASD (fasted) group.

e *p* < 0.01 compared with the PTM-HF-TPGS ASD (fasted) group.

f *p* < 0.05 compared with the reference (fed) group (using Duncan’s method).

The impact of food on the bioavailability of PTM formulations is presented in Figure 10B. A positive food effect was observed for the crystalline drug formulation where administration of the reference formulation in the fed state led to a significantly higher *C*_max_ and AUC than that observed for the fasted state (Table 3). Delayed drug transit from the stomach to the small intestine due to the presence of food presumably accounts for the absorption lag time and longer *T*_max_ observed in the fed state. For the ASD formulations, food had a similar impact on the lag time (Figure 10B). However, the much longer *T*_max_ and median retention time (MRT) observed in monkeys that were administered the ASD formulation with food suggests that drug release was slow and prolonged. Importantly, the ASD formulation was found to have a negative food effect with a significantly lower *C*_max_ and lower AUC than in the fasted state (*p* < 0.05). Thus, the HPMCAS-HF ASD showed enhanced bioavailability in the fasted state relative to the reference product but showed a negative food effect. In contrast, the reference product showed a positive food effect. However, there was a large variation between individual monkeys (Figure S11), and differences between groups were not always statistically significant (Table 3).

## 4 Discussion

### 4.1 Drug Crystallization Tendency

PTM is a 4-nitroimidazole derivative and is a structural analog of another recently approved TB therapy, delamanid. PTM has low aqueous solubility and low oral bioavailability,^9^ making it of interest for ASD formulation approach. Delamanid is commercially available as an ASD formulation,^46^ whereas PTM is in crystalline form in the approved product. Both of these drugs are low *T*_g_ compounds (∼10 °C for PTM and ∼43 °C for delamanid^47^) and are rapid crystallizers (class I),^48^ whereby the neat drugs exhibit poor glass stability. Reduced glass transition temperatures of PTM and delamanid (calculated as *T*_g_/*T*_m_)^25^ are 0.67 and 0.64, respectively. In aqueous solution, a similar pattern of rapid crystallization is observed for both drugs, with nucleation induction times of only a few minutes at supersaturations close to the amorphous solubility. However, while multiple polymers, including HPMCAS, HPMCP, and PVPVA, were found to effectively stabilize supersaturated solutions of delamanid,^47^ PTM was noted herein to be much more difficult to maintain as a supersaturated solution. Only HPMCAS-HF was able to delay PTM crystallization at supersaturation ratios of 5–10 (Figure 1 and Table S1). Furthermore, above the amorphous solubility, where a second, drug-rich phase of PTM is present, even HPMCAS-HF was unable to delay crystallization for longer than a few minutes (Figure S3). The high crystallization tendency of PTM can be attributed to several factors including its low *T*_g_, low MW, and relatively linear structure.^23−25^

Fabrication of ASD with a suitable polymer is a common strategy to inhibit amorphous drug crystallization. This approach was successful for PTM with HPMCAS-HF, yielding ASDs that were physically stable under ambient conditions for several months (Figure S12), in comparison to neat amorphous drug, which crystallized within a few minutes under the same conditions (Figure S2). However, recent studies have highlighted that solid-state stability and solution crystallization studies may not adequately predict the complex landscape of phase transitions that can occur in in vitro conditions that mimic gastrointestinal transit.^16,29,49^ This has led to the increased utilization of more complex in vitro testing conditions, including two-stage dissolution, where the ASD is first evaluated under conditions simulating gastric media, followed by transfer to fluids simulating intestinal conditions.^47,49−51^ In particular, it has been recently highlighted that immersion in gastric conditions, where enteric polymers such as HPMCAS are insoluble, can yield insights into important patterns of phase behavior.^29^ Thus, for weakly basic drugs formulated with an enteric polymer, it has been noted that the gastric pH influences both the extent of release from the ASD as well as the tendency of the drug to undergo crystallization on the matrix surface.^29,50−52^ The release extent variation has been rationalized based on the pH-dependent solubility of the basic drug,^50−52^ while the matrix surface crystallization under low pH conditions likely reflects the reduced ability of the polymer to act as a crystallization inhibitor at a lower pH when in un-ionized form.^29^

Based on the screening data of crystallization inhibition effectiveness, HPMCAS-HF was the only realistic polymeric candidate for formulating ASDs of PTM. Downsides of this particular polymer are its high reported threshold dissolution pH of >6.5^35^ and a relatively hydrophobic chemistry, which in combination may lead to slow and incomplete drug release in vivo. Indeed, during two-stage release testing, incomplete release was observed (Figure 3B). Out of the various strategies evaluated to improve the release from HPMCAS-HF dispersions, while concurrently maintaining supersaturation duration, surfactant (TPGS) inclusion and addition of a basic compound to the ASD were found to be successful based on in vitro evaluation. Addition of a base likely results in polymer salt formation and was recently found to enhance the dissolution rate of HPMCP.^28^ However, for the formulations containing a base, agglomerates were observed following tablet disintegration. Consequently, the release extent of this formulation was susceptible to the hydrodynamic conditions of the release test and was also negatively impacted by the compaction process, where gelation hindered disintegration and delayed release (Figure S9).

In contrast, combining different grades of HPMCAS, while allowing for more rapid release in some systems, was unsuccessful at achieving sustained and high levels of supersaturation (Figure 2C,D). This was attributed to inefficient crystallization inhibition during the gastric immersion stage, leading to a greater extent of surface crystallization during gastric immersion (Figure S4) and reduced drug release (Figure 2C,D). Furthermore, HPMCAS-HF was also found to play a key role in preventing drug crystallization from the supersaturated solution formed after drug release (Figure 2B), and thus, a reduction in the total HPMCAS-HF concentration in solution, resulting from the “dilution” by the other grades (Table S2), was also detrimental to the in vitro performance. It is clear from the in vitro observations that PTM is a difficult compound to formulate as an ASD due to its high propensity to crystallize during the gastric immersion stage, and from the supersaturated solution generated following polymer dissolution.

### 4.2 Oral Bioavailability and the Impact of Food

Enteric polymers have been frequently used in ASD formulations and provide enhancements to drug amorphous stability to crystallization, release, and ultimately bioavailability.^53−56^ For example, the bioavailability in cynomolgus monkeys given a single oral dose of an ASD of posaconazole with HPMCAS-MF (Noxafil, Merck, US) was higher than that observed with the crystalline suspension formulation.^55^ Hot-melt extruded ziprasidone ASD with a combination of HPMCAS-HF and Plasdone-S630 (20:56.66:59.18 mass ratio) was also found to improve bioavailability in beagle dogs, in comparison to the commercial nanocrystal formulation (Zeldox, Pfizer, USA).^56^ For pretomanid and HPMCAS-HF ASD, enhanced bioavailability was observed in the fasted state, as compared to the crystalline formulation (Figure 10A). Interestingly, although manipulation of the ASD formulation through addition of either TPGS or tris led to improved drug release in vitro, this did not translate to the in vivo situation, in particular, for the HF-Tris formulation. While the binary ASD and that containing TPGS gave similar in vivo profiles, the ASD containing tris was markedly different. In particular, *T*_max_ was notably prolonged in this formulation, suggesting a much slower drug release and subsequent absorption. This can be attributed to the gelation tendency of this formulation, which was masked in the in vitro study when a high stirring rate was used. Lowering the stirring rate was important to enable differentiation between the various ASD formulations (Figure 7B,D), highlighting that our initial in vitro testing conditions were not optimized to mimic the physiological conditions important in impacting release from the ASD. Based on the in vivo outcome, it appears that gelation of susceptible formulations may be more likely to occur in vivo than in a relatively well-stirred dissolution vessel.

Drug–food interactions and their impact on bioavailability are important considerations in drug development. It is important to understand the mechanisms involved in drug–food interactions to achieve better predictions of possible clinical impacts.^57^ Omachi et al. reported that drugs with high log *P* and low intestinal solubilities will likely show a positive food effect with improved absorption in the fed state.^58^ This is due to the fact that poorly soluble BCS class II drugs are often solubilized by food and bile components, including bile salts, lecithin, and fatty acids.^59^ For pretomanid, a considerably higher crystalline solubility was observed in FeSSIF (Table 2), and this translated into a higher bioavailability of the crystalline reference product for the fed state (in monkeys, Figure 10B and Table 3), consistent with observations in a clinical study.^8^ Food increased the *C*_max_ and AUC_0-inf_ values by 76 and 88% in humans,^8^ while in this study using monkeys, the difference in these parameters were 23 and 49%, respectively.

However, the influence of food on drug absorption can vary for different formulations.^57^ There are very few available reports discussing the impact of prandial state on ASDs formulated with an enteric polymer. Both posaconazole and ziprasidone were found to have positive food effects when dosed as crystalline formulations, while ASDs of these drugs were not markedly affected by food in human (for posaconazole)^60^ and dog models (for ziprasidone).^56^ One important consideration is that, due to the pH-dependent solubility of the polymer, drug absorption from a dosage form with an enteric polymer can be impacted not only by the delayed gastric emptying caused by the fed state but also by a delayed intestinal release whereby formulations need to reach the region of the gastrointestinal (GI) tract where the pH is sufficiently high for the polymer to commence dissolution and allow for drug release.^57,61^ Thus, it is well established that food ingestion results in delayed and more variable absorption of aspirin from enteric-coated tablets.^61,62^ In addition, variations in drug absorption between individual subjects, attributed to pH variation in the GI tract were observed for enteric-coated tablets of an investigational compound when HPMCAS-MF was used as the enteric polymer.^63^ This pH variation might also contribute to the large variation of drug absorption observed between monkeys administered PTM-HF ASD tablets, especially in the fed state (Figure S11). Moreover, the low-fed-state AUC observed with PTM-HF ASD tablets suggests that drug release is likely impeded due to the low polymer solubility at the lower-fed-state intestinal pH, as illustrated by in vitro release testing (Figure 8).

Moreover, differences between species in GI tract characteristics, such as pH, GI transit time, and mobility, can also affect drug absorption from formulations containing components with pH-dependent solubility.^64^ In the presence of food, the cynomolgus monkey was observed to have a longer and more variable gastric emptying time (up to 5 h) as well as a lower small intestinal pH (around 5–6) compared to a human or a dog.^64−66^ Any pH variations may lead to notable changes in the drug crystallization tendency and release from ASDs formulated with an enteric polymer. Kohri et al. indicated that ASDs of albendazole with HPMC and HPMCP-55 achieved 100% bioavailability in rabbits with normal gastric acidity (pH ∼ 1), while bioavailability was reduced to 62% when the ASD was administered under low gastric acidity conditions (pH ∼ 5).^67^ All grades of HPMCAS polymer have a p*K*_a_ of ∼5,^45^ but the succinoyl content impacts the polymer dissolution pH threshold.^68^ HPMCAS-HF, which has the lowest succinoyl content of the available HPMCAS grade, requires a higher pH to commence dissolution, which may lead to an extended time lag and reduced dissolution rate.^68^ Herein, the reduced in vivo absorption of the ASD formulation in the fed state is attributed to the slow polymer dissolution rate due to the lowered intestinal pH in the fed-state monkey. It is further likely that PTM underwent matrix crystallization due to the extended time in the ASD formulation prior to release, as seen in Figure S10.

## 5 Conclusions

The high crystallization tendency of PTM and the paucity of effective polymeric crystallization inhibitors limited polymer choice for the fabrication of ASDs; HPMCAS-HF was the only viable choice from a crystallization inhibition perspective. The in vitro drug release in simulated GI fluids could be improved by generating ternary ASDs containing TPGS or a basic excipient. ASDs demonstrated enhanced oral bioavailability compared to a reference formulation containing crystalline drug when dosed to cynomolgus monkeys under fasting conditions, with the binary ASD tablets showing the best performance. While drug absorption from the reference formulation showed a positive food effect, the binary ASD displayed sustained release, and a reduced extent of absorption in the fed state. The poor translation between in vitro drug release and in vivo pharmacokinetics suggests that HPMCAS-HF-based ASDs of PTM may be highly sensitive to intestinal pH conditions, and that drug crystallization in the ASD matrix is exacerbated due to delayed release from the formulation. Further studies are required to provide an improved understanding of food impact and to develop an optimized methodology to correlate the in vitro observations to the in vivo outcomes for ASDs based on enteric polymers.

## Supporting Information Available

Solubility of drug in the presence of surfactant or in FaSSIF V1; rapid crystallization tendency of amorphous drug observed by DSC; induction time of PTM as a function of drug concentration and the impact of polymer; drug crystallization and agglomeration of 10 and 20% DL ASDs during dissolution; impact of the TPGS ratio on the glass transition temperature of ASDs; basic additives used in the study; impurities in ASDs confirmed by ^19^F NMR spectra; dissolution of reference tablets in biorelevant media; agglomeration of PTM-HF-Tris tablets; drug crystallization in low intestinal pH; individual variations of drug absorption in vivo; and physical stability of ASDs (PDF)

## Abbreviations

ASD: amorphous solid dispersion
AUC: area under the curve
ANOVA: analysis of variance
BCS: biopharmaceutics classification system
CAP: cellulose acetate phthalate
DL: drug loading
DMEA: 2-dimethylaminoethanol
DSC: differential scanning calorimetry
EDR: extensively drug-resistant
GI: gastrointestinal
HPLC: high performance liquid chromatography
HPMC: hydroxypropyl methylcellulose
HPMCAS: hydroxypropyl methylcellulose acetate succinate
HPMCP: hydroxypropyl methylcellulose phthalate
LC-MS/MS: liquid chromatography tandem mass spectrometry
LLPS: liquid–liquid phase separation
MCC: microcrystalline cellulose
MDR: multidrug resistant
MDEA: *N*-methyldiethanolamine
MeOH: methanol
MW: molecular weight
NMR: nuclear magnetic resonance
PB: phosphate buffer
PK: pharmacokinetics
PLM: polarized light microscopy
PTM: pretomanid
PVPVA: polyvinylpyrrolidone/co-vinyl acetate
PXRD: powder X-ray diffraction
SEM: scanning electron microscopy
SLS: sodium lauryl sulfate
SR: supersaturation ratio
SSG: sodium starch glycolate
TB: tuberculosis
TPGS: vitamin E tocopheryl polyethylene glycol succinate
UV: ultraviolet.
