# Supplementary material for: Fed- and Fasted-State Performance of Pretomanid Amorphous Solid Dispersions Formulated with an Enteric Polymer
Source: Mol Pharm. 2023 May 23;20(6):3170–86. doi: 10.1021/acs.molpharmaceut.3c00174 (PMC10245392; doi:10.1021/acs.molpharmaceut.3c00174)
Supplement: Supplementary file 1 — mp3c00174_si_001.pdf [file mp3c00174_si_001.pdf]

Supplementary Information

Fed and fasted state performance of pretomanid amorphous solid dispersions formulated with an enteric polymer

Hanh Thuy Nguyen[1], Tu Van Duong[1], Sarah Jaw-Tsai[2], Rebecca Bruning-Barry[3], Poonam Pande[4], Rajneesh Taneja[4], Lynne S. Taylor[1]*

[1] Department of Industrial and Physical Pharmacy, College of Pharmacy, Purdue University, West Lafayette, Indiana 47907, United States

[2] Sarah Jaw-Tsai Consulting Services, 12279 Skyracer Drive, Las Vegas, NV 89138, United States

[3] RTI International, Global Health Technologies Program, Research Triangle Park, NC 27704, United States

[4] Global Alliance for TB Drug Development (TB Alliance), 80 Pine Street, 20th Floor, New York, NY 10005, United States

* Corresponding author. E-mail: lstaylor@purdue.edu. Tel: +1 (765) 496-6614. Fax: +1 (765) 494-6545.

Supplementary data

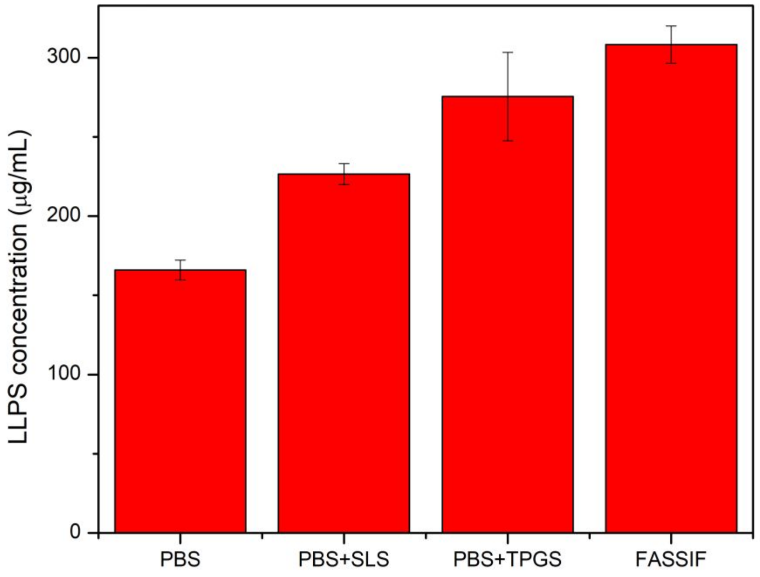

Figure S1. LLPS concentration of PTM in PBS pH 6.5 with or without surfactant (SLS or TPGS at 200 µg/mL) or SIF powder.

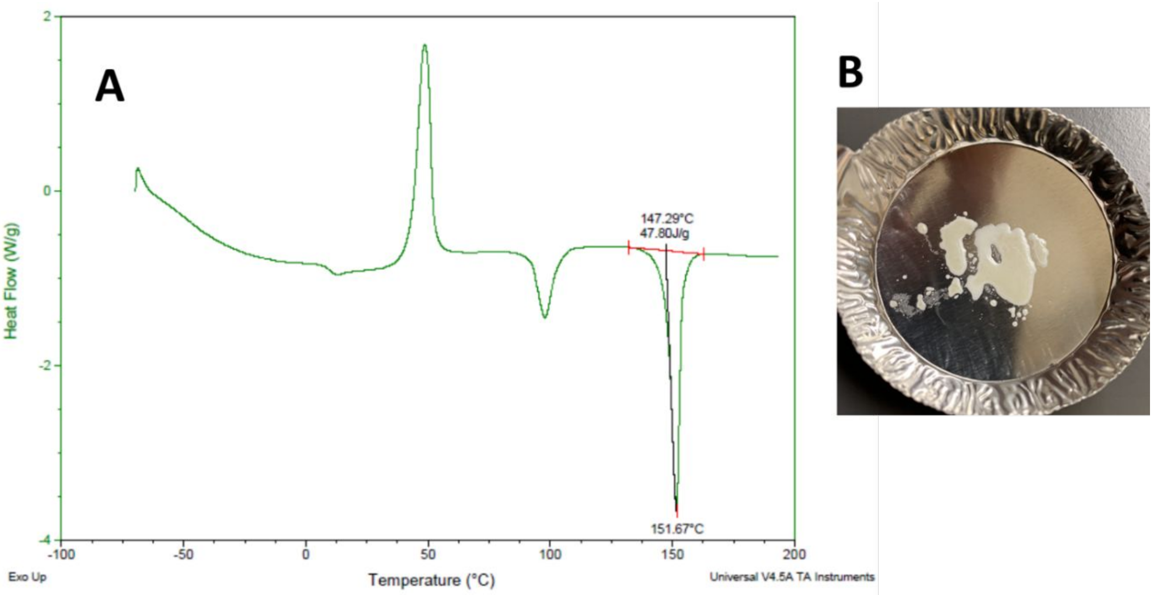

Figure S2. (A) Rapid crystallization of glassy PTM obtained by quenching the melt in liquid nitrogen based on the DSC thermogram and (B) Crystallization of glassy PTM after a few minutes at room temperature.

Table S1. Induction time of PTM at 160 μg/mL in phosphate buffer pH 6.5 or FaSSIF V1

Medium		Induction time (min)
No polymer	Phosphate buffer pH 6.5	1.4 ± 0.2
	FaSSIF V1	0.9 ± 0.3
1 mg/mL HPMCAS-HF	Phosphate buffer pH 6.5	43.8 ± 1.3
	FaSSIF V1	77.6 ± 13.6

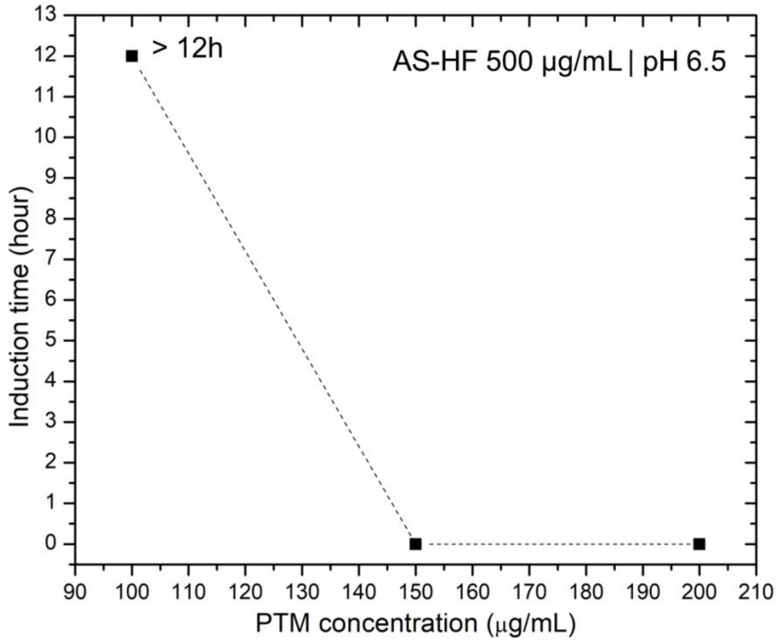

Figure S3. Induction time of PTM as a function of drug concentration in phosphate buffer pH 6.5 with 500 μg/mL HPMCAS-HF

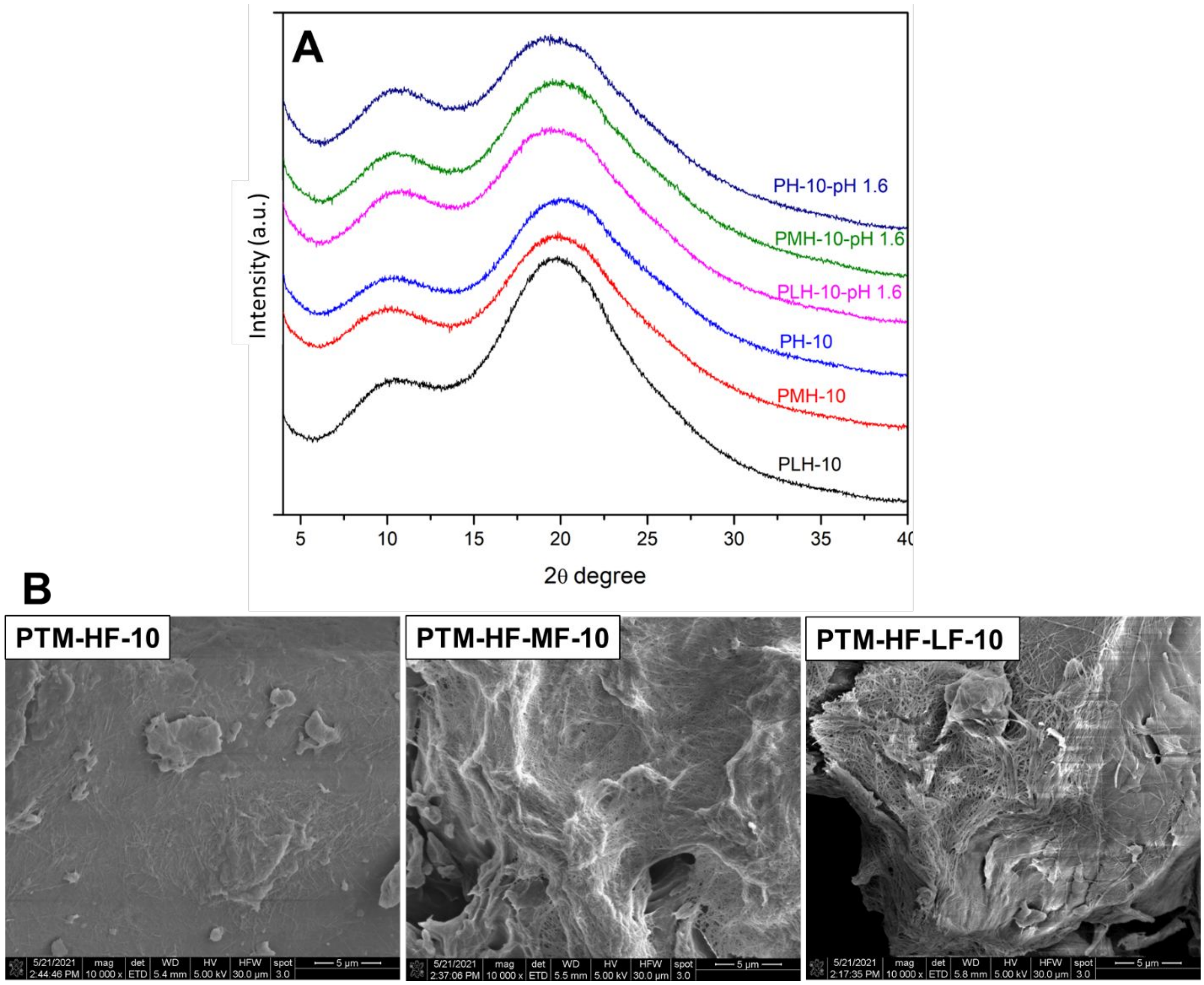

Figure S4. Drug crystallization in 10% DL ASDs after incubation in HCl solution pH 1.6 for 1h: (A) PXRD analysis and (B) SEM images.

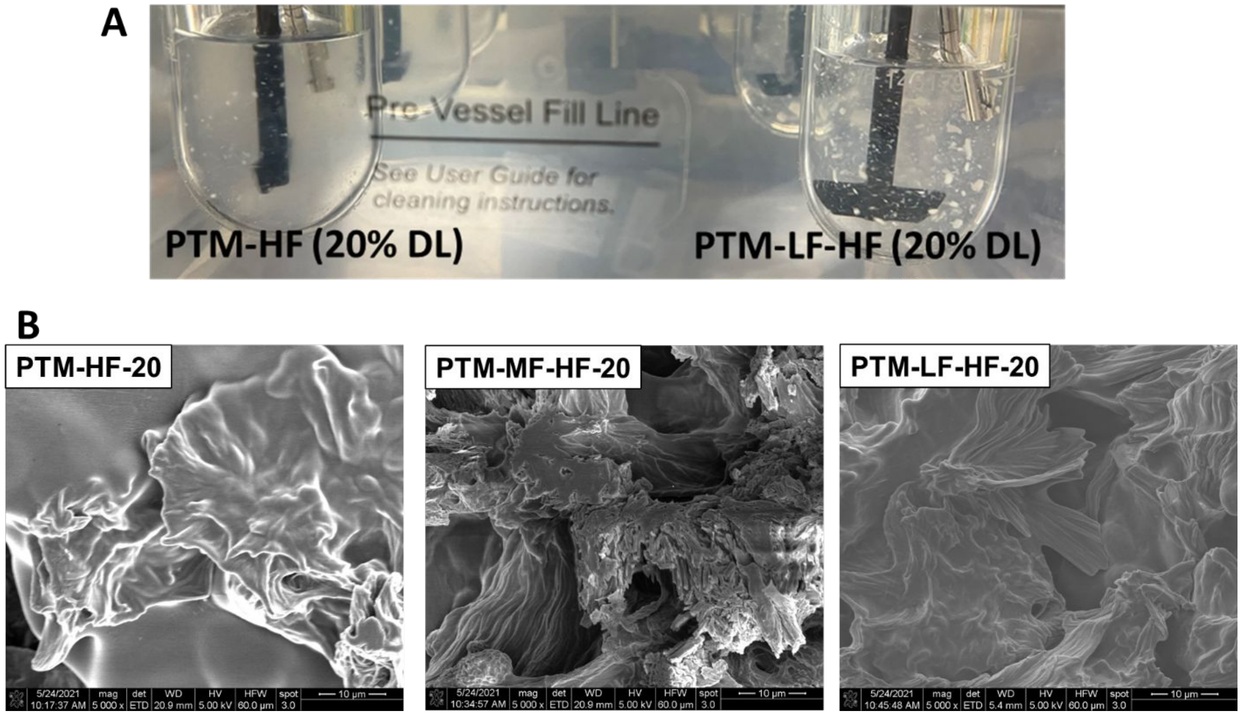

Figure S5. (A) Agglomeration of undissolved ASD (20% DL) particles and (B) Morphology of undissolved ASD particles after dissolution in FaSSIF for 60 min.

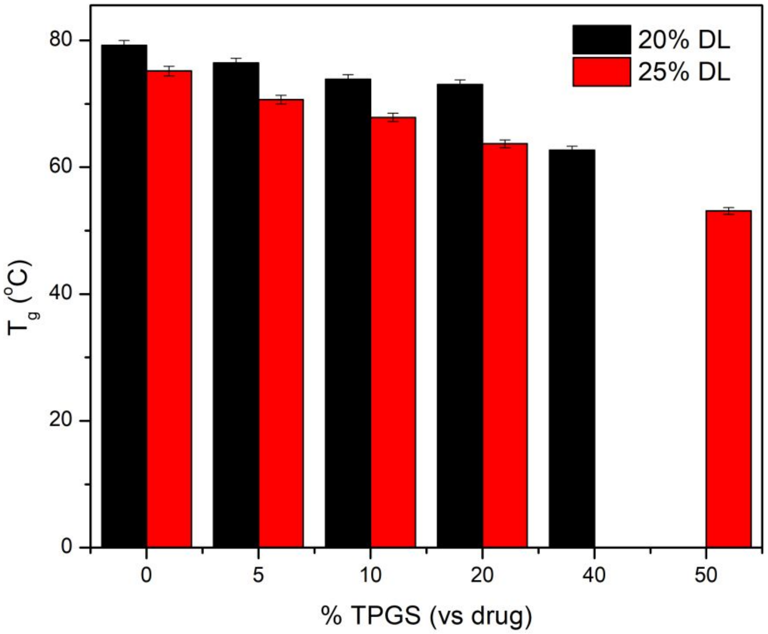

Figure S6. Impact of TPGS ratio on glass transition temperature of PTM-HF-TPGS ASDs.

Table S2. Basic additives incorporated into PTM-HF ASDs at 20% DL

Basic compound	Molecular structure	pKa*	MW (g/mol)	ASD composition (mg)		
				PTM	Polymer	Base
HF only		-	-	400	1600.0	
Triethylamine	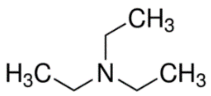	10.64 [1]	101.19	400	1481.3	118.7
2-Dimethylaminoethanol (DMEA)	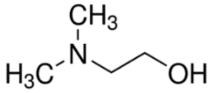	9.23 [2-3]	89.14	400	1494.5	105.5
N-Methyldiethanolamine (MDEA)	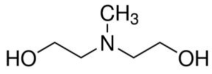	8.54 [1]	119.16	400	1462.0	138.0
Triethanolamine	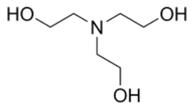	7.73[1]	149.18	400	1430.9	169.1
Tris base	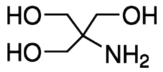	8.1 [4]	121.14	400	1459.9	140.1
Meglumine	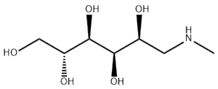	9.58 [5]	195.21	400	1385.7	214.3
Ammediol	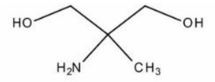	8.84 [3, 6]	105.14	400	1477.0	123.0
Proline sodium	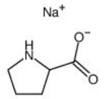	10.6	137.11	400	1441.3	158.7

**The highest pKa value at 25°C*

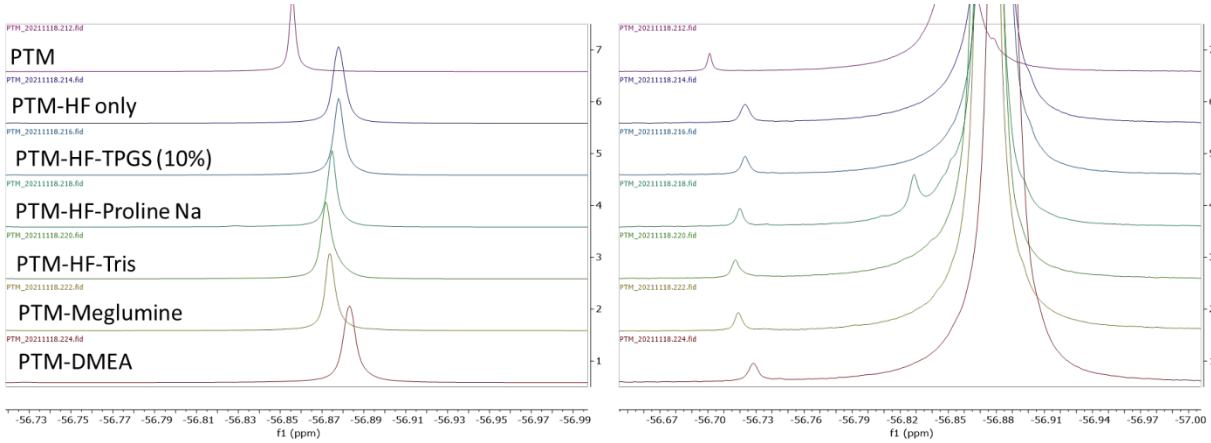

Figure S7. ^{19}F-NMR spectra of PTM and PTM ASDs at 20% DL.

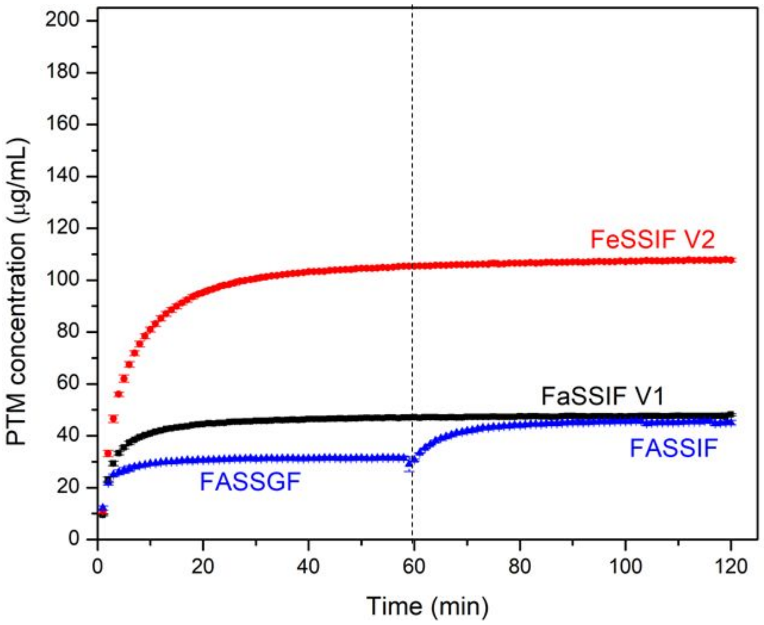

Figure S8. Dissolution of reference PA-824 tablets in biorelevant media (maximum concentration of 200 µg/mL) at 150 rpm stirring speed with the dotted line indicating the transition point from gastric to intestinal media (blue) or single stage release in FaSSIF V1, pH 6.5 (red) or fed state simulated intestinal fluid (FeSSIF V2, pH 5.8) (black).

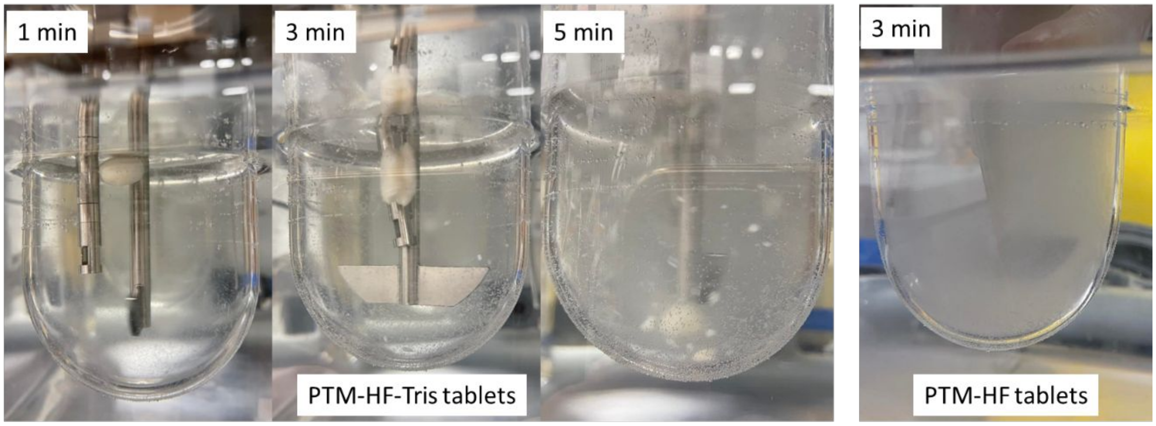

Figure S9. Slow disintegration of PTM-HF-Tris tablets during dissolution in FeSSIF V2, pH 5.8 at 75 rpm. Disintegration of PTM-HF tablets in same conditions is shown in the far right photo as a reference.

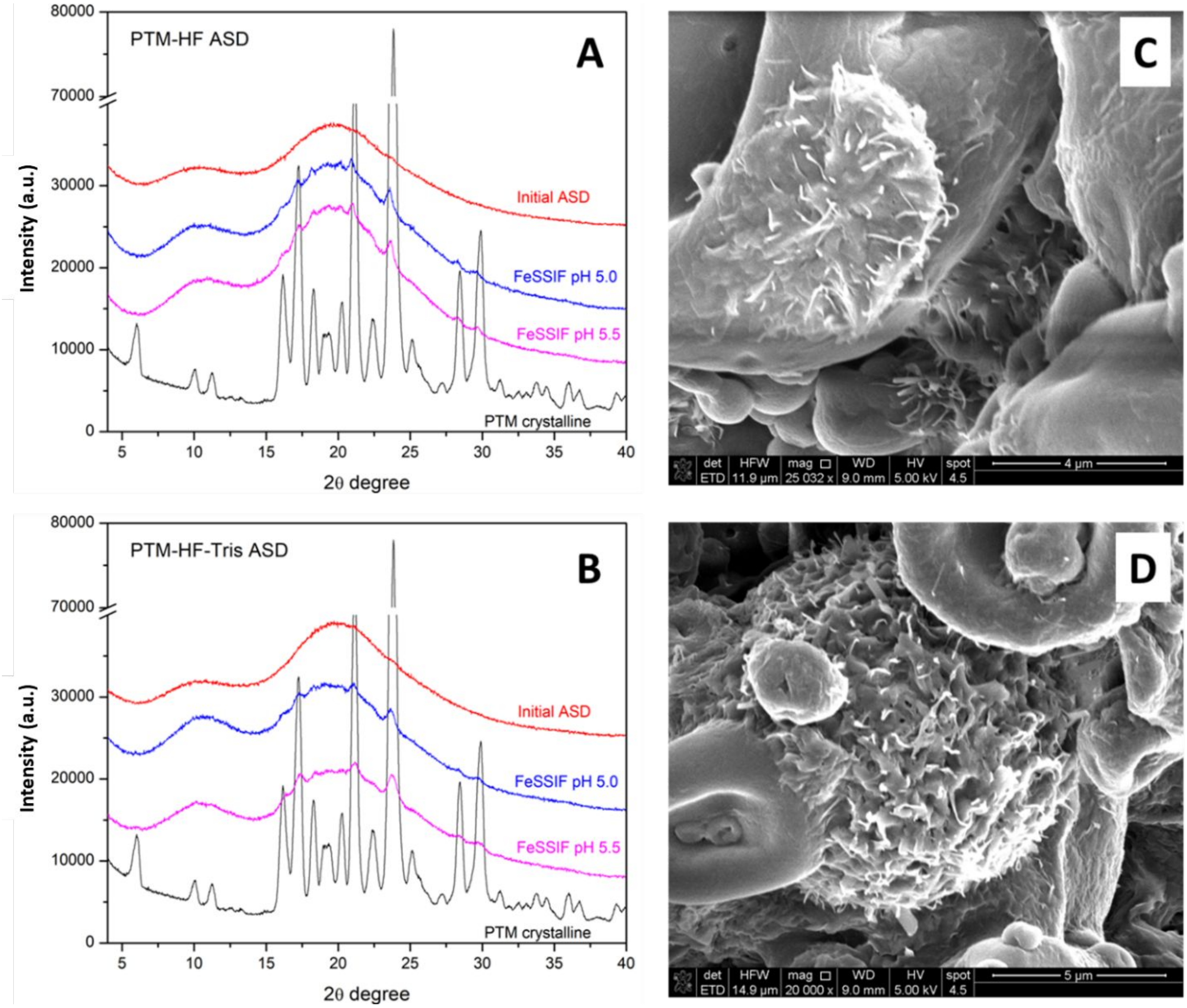

Figure S10. Matrix crystallization in simulated intestinal media with different pH conditions observed by (A-B) PXRD and (C-D) SEM for (A, C) PTM-HF ASD and (B, D) PTM-HF-Tris ASD at 20% w/w DL.

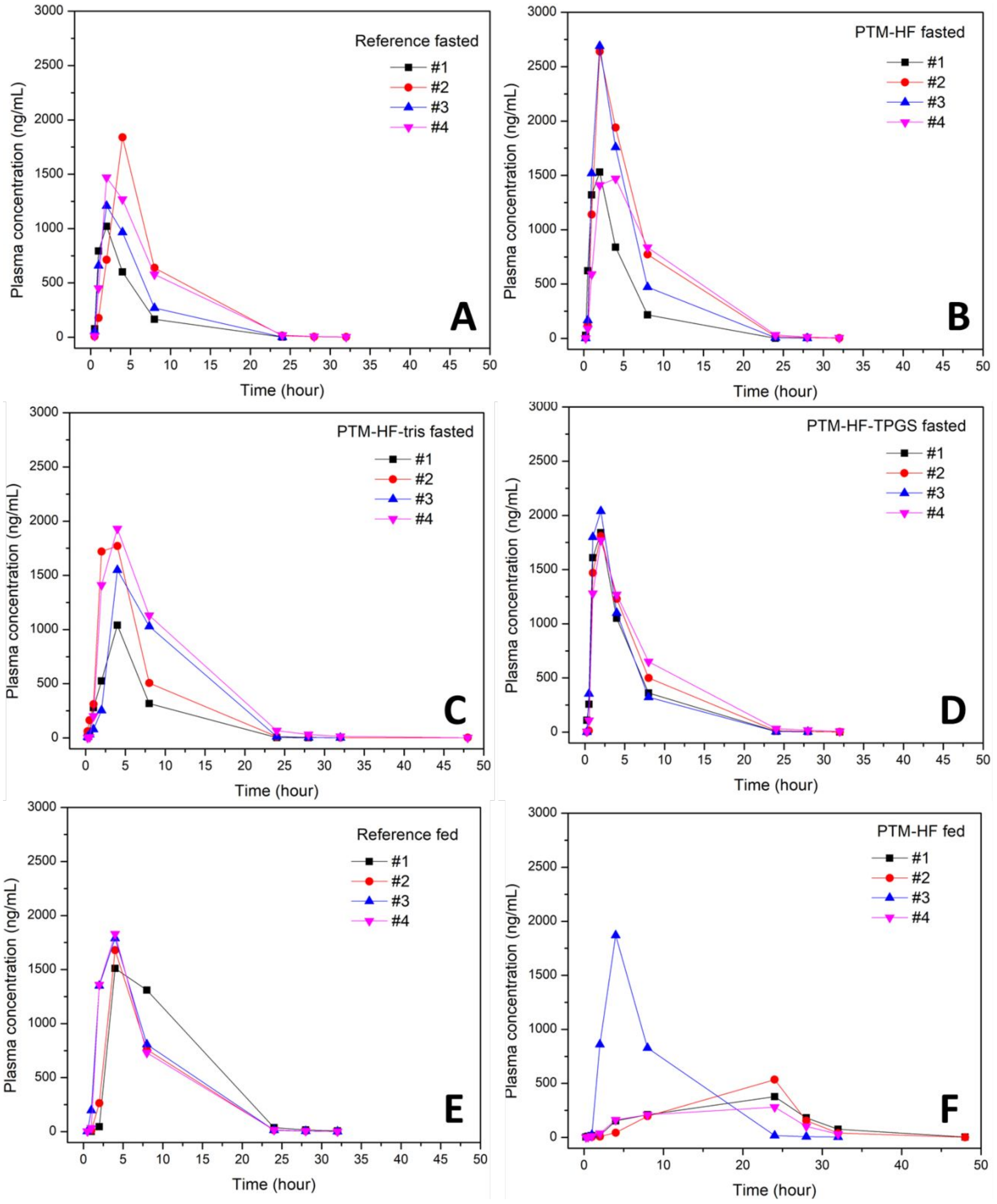

Figure S11. Plasma concentration in individual monkeys administered (A; E) reference formulation, (B; F) PTM-HF ASD tablet, (C) PTM-HF-Tris tablet, or (D) PTM-HF-TPGS tablet under (A-D) fasted or (E-F) fed conditions.

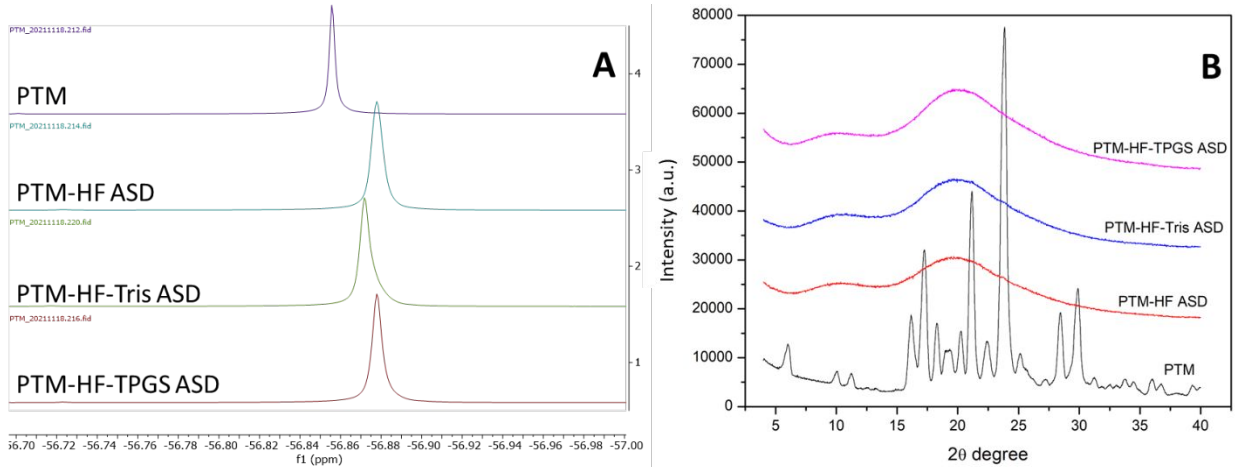

Figure S12. (A) ^{19}F-NMR spectra of PTM and PTM ASDs after one-month storage at room temperature and (B) PXRD of ASDs after three-month storage at room temperature.

References

1. Rayer, A. V.; Sumon, K. Z.; Jaffari, L.; Henni, A., Dissociation Constants (pKa) of Tertiary and Cyclic Amines: Structural and Temperature Dependences. *J. Chem. Eng. Data* **2014,** *59* (11), 3805-3813.
2. Littel, R. J.; Bos, M.; Knoop, G. J., Dissociation constants of some alkanolamines at 293, 303, 318, and 333 K. *J. Chem. Eng. Data* **1990,** *35* (3), 276-277.
3. Eshaghi Gorji, Z.; Ebrahimpoor Gorji, A.; Riahi, S., A structure-property model for the prediction of pKa values of different amines in the CO2 capture process of concern to the prediction of thermodynamic properties. *Chem. Eng. Res. Des.* **2022,** *180*, 13-24.
4. Noroozi, J.; Smith, W. R., Prediction of Alkanolamine pKa Values by Combined Molecular Dynamics Free Energy Simulations and ab Initio Calculations. *J. Chem. Eng. Data* **2020,** *65* (3), 1358-1368.
5. Bazeed, A. Y.; Essa, E. A.; Nouh, A.; El Maghraby, G. M., Co-processing of nateglinide with meglumine for enhanced dissolution rate: in vitro and in vivo evaluation. *Drug Dev. Ind. Pharm.* **2020,** *46* (10), 1676-1683.
6. Joback, K. G.; Heberle, J. R.; Bhown, A. S., Influence of pKa and Amine Structure on Energy Consumption of Post-combustion CO2 Capture Processes. *Energy Procedia* **2017,** *114*, 1689-1708.
